# Thin-Layer Behavior in Carbon Nanopipettes. Understanding the Iontronic-Electronic Contributions

**DOI:** 10.1021/acs.analchem.5c02834

**Published:** 2025-08-05

**Authors:** Gregorio Laucirica, Gastón A. Crespo, María Cuartero

**Affiliations:** † UCAM-SENS, Universidad Católica San Antonio de Murcia, 16728 UCAM HiTech, Avda. Andres Hernandez Ros 1, 30107 Murcia, Spain; ‡ Department of Chemistry, School of Engineering Science in Chemistry, Biochemistry and Health, 7655 KTH Royal Institute of Technology, Teknikringen 30, SE-114 28 Stockholm, Sweden

## Abstract

Nanopipettes with carbon-coated inner surfaces (carbon nanopipettes, CNPs) have attracted considerable attention due to their exceptional sensitivity and potential in electroanalytical applications. The nanoconfinement of the sample solution within the CNP facilitates a thin-layer electrochemical regime, in which ion and electron transferences are inherently coupled. This feature allows exhaustive oxidation/reduction of certain analytes within typical electroanalytical time scales, offering unprecedented opportunities for nanoscale sensing. Despite this promising advantage, a detailed understanding of how measurement dimensions and experimental conditions influence key electrochemical responses remains significantly underexplored. Effectively, conventional electrochemical methods frequently struggle with decoupling ionic and redox contributions, which are critical for understanding the performance toward optimal exploitation. For the first time, cyclic voltammetry (CV), numerical simulations, and electrochemical impedance spectroscopy (EIS) are combined to systematically investigate the interplay between ion transport and electron transfer in the electrochemical behavior of CNPs. CV experiments were used to assess essential parameters under varying electrolyte compositions, solution depths, and scan rates, achieving signal-to-noise ratio enhancements of over 10-fold and submicromolar detection of the redox couple at the rationalized conditions. Complementarily, it is demonstrated that EIS can resolve the nanofluidic behavior by deconvoluting iontronic and electronic contributions, opening an option to be investigated more extensively in future research. The present study not only provides insights into the unique thin-layer electrochemical behavior of CNPs but also establishes the feasibility of simultaneously obtaining iontronic and electronic information with a single setup. This dual capability is poised to advance both related applications, e.g., sensing, (bio)­catalysis, imaging, and fundamental directions in nanoelectrochemistry.

## Graphical abstract

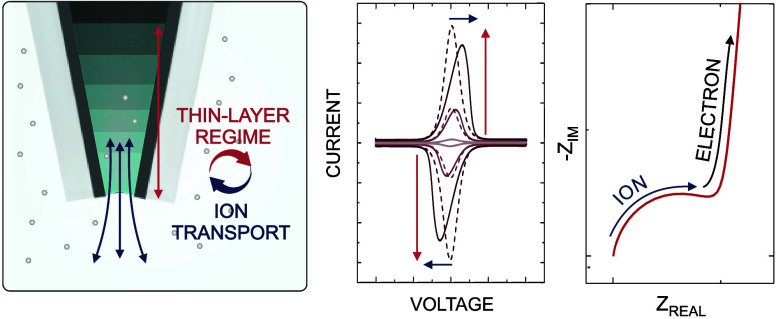

## Introduction

Gradual technological advancements have promoted the development of specific subfields within nanotechnology, such as nanofluidics, prone to be advantageously integrated toward a nanoelectrochemistry vision.^1,2^ Effectively, in combination with electrochemical techniques, nanofluidic devices display great potential for creating (bio)­sensing and (bio)­catalysis platforms, energy conversion systems, and integrated circuits.^3–6^ Specifically, two types of signals–iontronic and electronic–can be recorded.^5^ In iontronic signals, ions act as carriers, and the signal is determined by the ion flux through the nanostructure conforming the electrode, providing hence information about surface chemistry and mass transport. This has been demonstrated in the form of nanotips, nanopores, and nanochannels. Instead, when electronic signals are obtained, the nanofluidic device typically serves as the working electrode (WE), and the signal corresponds to redox reactions occurring on its surface (i.e., pure electron transfer processes). The combination of iontronic and electronic signals has shown exceptional capabilities for high-resolution imaging of biomaterials,^7^ and sensing.^8^

Glass nanopipettes with inner conducting walls based on thin carbon layers (carbon nanopipettes, or CNPs) are nanoelectrochemical devices that have gained attention due to their sensitivity and potential for single-cell measurements.^5,9–11^ Its architecture uniquely combines a nanostructure electrode in contact with the sample confined within its interior. On one hand, the electrical properties of the thin carbon layer generate a WE that enables electrochemical experiments under nanoconfinement conditions.^12^ On the other hand, as the surface-to-volume ratio is significantly enlarged, the behavior of CNPs (and certain micropipettes) differs from typical observations in macroscopic electrodes. For instance, Kashyap et al. demonstrated that carbon fiber microelectrodes exhibit significantly different electrochemical behavior compared to bulk systems when experiments were conducted in volumes on the order of picoliters.^13^ Then, pioneering studies by Mirkin and colleagues have shown that CVs of redox probes using CNPs with volume-defined cavities exhibit thin-layer properties, enabling rapid coulometric determination of redox compounds.^14^ Since then, CNPs have been widely employed for sensing purposes.^10,15,16^ Undoubtedly, the described finding represents a promising prospect for using CNPs in calibration-free quantitative analysis, being this feature not deeply explored at the time of writing. In contrast, research efforts have been focused on understanding the fundamental electrochemical phenomena within the confined space of CNPs.^14,17^ It has been established that the electrochemical response provided by the CNP is determined by the compromise of heterogeneous redox reactions on the carbon surface and ion transport between the confined and the bulk sample across the CNP tip. For this reason, the CV shape is drastically affected by parameters such as scan rate (*v*) or salt concentration. To the best of our knowledge, a systematic understanding of conditions influencing the thin-layer behavior remains lacking, while crucial for expanding the horizon of the CNPs.

Comprehending the complex interplay between ion and electronic transfers in CNP-based systems is challenging. Traditional electrochemical techniques relying on direct current measurements often face drawbacks in separating these contributions. Alternating current-based methods, e.g., EIS, can provide powerful insights into the underlying working mechanism by enabling the decomposition of processes occurring at different time scales, such as ion transport and redox transfer, within a single measurement.^18–20^ The use of EIS with nanofluidic devices has attracted considerable attention in the past few years, providing essential information on ion transport characteristics, ion selectivity, and memristive properties of nanopipettes and nanoporous membranes.^21–23^ Noticeably, in those cases, the experiments involved purely iontronic responses in nonconductive nanofluidic devices without any redox contribution.

Herein, we report on a fundamental study of the thin-layer properties of CNPs under different conditions affecting the interconnected charge-transfer processes involved in the operation principle. Our investigation aims to uncover the phenomena that dictate electrochemical performance under nanoconfinement and provide insights into the thin-layer properties of the CNPs. For such a purpose, CV, EIS, and finite element simulations are utilized. The proposed approach has three main dimensions: (1) to highlight the largely untapped potential of EIS for characterizing the distinctive behavior of CNP-based electrodes; (2) to explore how thin-layer behavior, and key CV-derived parameters–such as peak current (*I*_*p*_), capacitive current (*I*_*c*_), and their ratio (*I*_*p*_/*I*_*c*_)–respond to variations in experimental conditions, including solution depth, concentration, and scan rate *(v)*; (3) to propose different strategies for optimizing the electrochemical behavior of the CNPs, with particular focus on thin-layer coulometry for sensing applications. By addressing these aspects, our work delivers valuable information into the intricate dynamics of nanoelectrochemistry, while identifying strategies to optimize the sensing performance of CNPs.

## Experimental Section

### Materials

Potassium chloride (99.5%, KCl), ferrocene methanol (97%), and K_4_Fe­[II]­(CN)_6_·3H2O (99%) were purchased in VWR chemicals. Tetrabutylammonium chloride (≥97.0%, TBACl), Ag wires (0.5 mm diameter) and sodium hypochlorite solution (6–14% chlorine) were provided by Merck. Quartz capillary tubes without filament (0.7 mm inner diameter, 1.0 outer diameter, and 10 cm length) were obtained from Sutter Instrument (Novato, CA). All the reagents were employed without any further treatment. All solutions were prepared in ultrapure water (18.2 MΩ cm at 25 °C, Milli-Q water system, Merck Millipore).

### Carbon Nanopipette Fabrication

Two kinds of nanopipettes were fabricated from quartz capillaries by employing a CO_2_ laser-based puller P-2000 (Sutter Instrument). The programs (heat, filament, velocity, delay, and pull) used for the fabrication were 700, 4, 60, 145, and 175 for Program 1 and 700, 4, 30, 130, and 90 for Program 2. Then, a carbon layer was deposited onto the inner surface of the glass nanopipettes by chemical vapor deposition (CVD).^11,12^ For this, the glass nanopipettes were exposed to a mixture of CH_4_:Ar 0.2:0.6 L min^–1^ for 3.5 min at 925 °C. More details about the protocols and microscopy characterization (Figure S1) are provided in Sections 1 and 2 of the Supporting Information.

### Electrochemical Setup

All electrochemical experiments were performed using a three-electrode setup consisting of a Pt rod (1.92 mm diameter, electroactive area >2 cm^2^, Metrohm Nordic AB, Sweden) as the counter electrode (CE) and a homemade Ag/AgCl wire as the reference electrode (RE). The inner surface of the CNP was used as the WE by inserting an Ag wire into the back of the capillary to create the necessary electrical connection between the nanotip and the potentiostat socket. The WE was the CNP fabricated following Program 1 unless otherwise indicated. The electrodes were connected to a potentiostat VIONIC (Metrohm) operated with the Intello 1.5 software. All the experiments were carried out inside a Faraday cage (Rittal, GmbH & Co. KG). CVs were typically recorded at 50 mV s^–1^ in the potential window between–0.2 and 0.6 V, otherwise mentioned. EIS experiments were performed varying the frequency from 1 Hz to 1 × 10^6^ Hz, recording ten points per decade and applying a sinusoidal perturbation with an amplitude of ± 10 mV, otherwise mentioned. The sinusoidal perturbation was superimposed on a given direct current potential (E_DC_). In all cases, experiments were conducted using freshly prepared solutions, obtained by weighing the reagents as received from the supplier. Details of the experimental setup and analysis are provided in Section 1 of the Supporting Information.

## Results and Discussion

### Voltammetric Response at Different Scan Rates

Figure 1a illustrates the experimental setup used for the electrochemical measurements, with a three-electrode arrangement. In the WE, the carbon layer only coated the inner glass surface without occluding the pipet orifice, generating hence open CNPs.^11,12^ In the initial voltammetric studies, we observed that open CNPs exhibited a time-dependent electrochemical response due to capillary action-driven changes in the pipet’s filling level with the sample solution. As discussed below, this effect is herein systematically studied using CV and EIS. Notably, when evaluating the impact of experimental variables other than the filling degree on the electron transfer-ion transport mechanism (e.g., redox probe or supporting electrolyte concentration), CNPs with a stable volume were used: volume stability was confirmed by achieving a consistent and reproducible CV.

**Figure 1:**
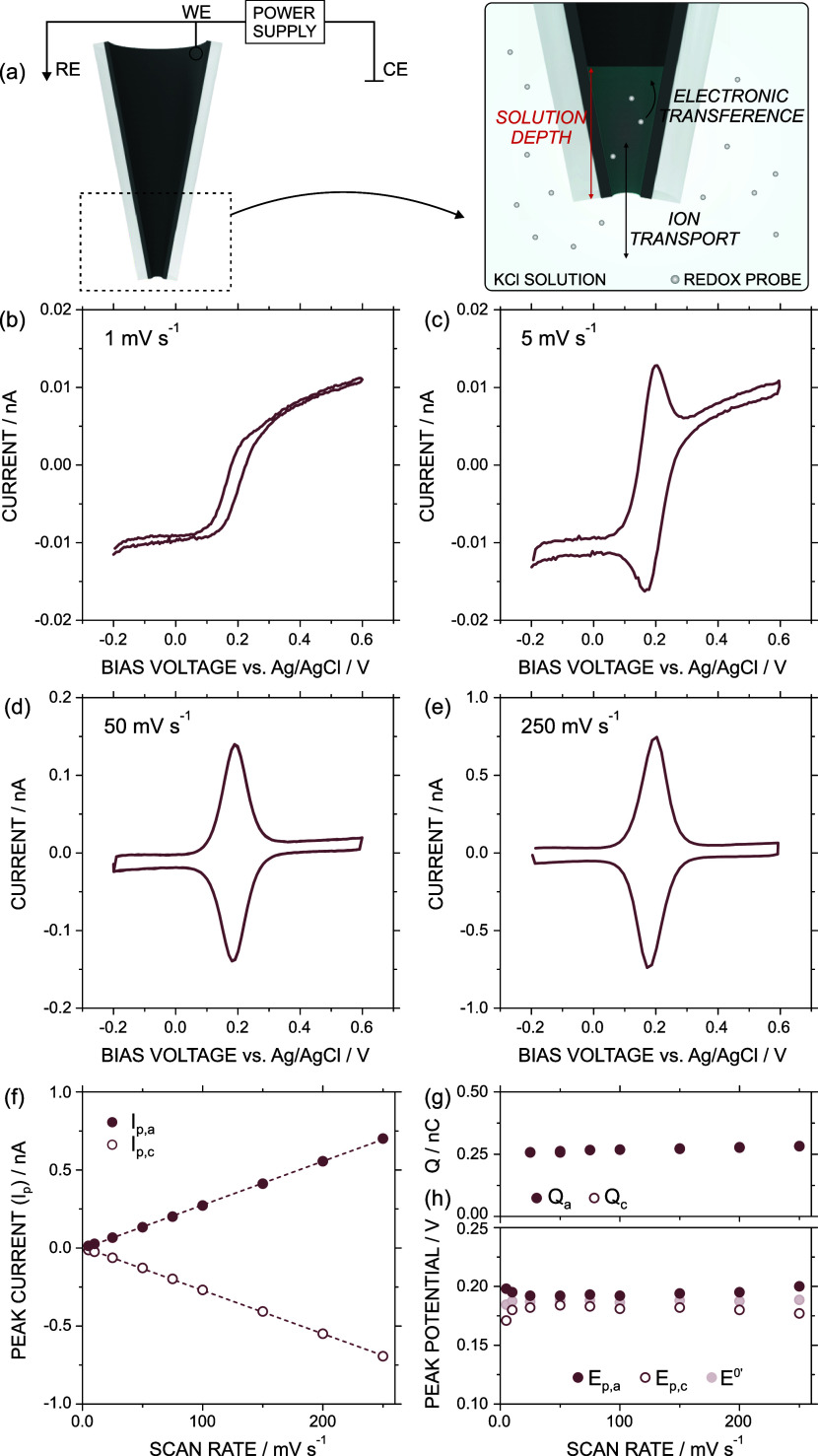
(a) Scheme of the experimental setup. (b–e) CV measurements at different scan rates: 1, 5, 50, and 250 mV s^–1^, respectively. Sample solution: 0.3 M KCl and 7.7 × 10^–4^ M K_4_Fe^[II]^(CN)_6_. (f) Peak current vs scan rate. (g) Voltammetric charge Q vs scan rate. (h) Peak potential *E*_p_ vs scan rate. Subindex “c” and “a” refer to the cathodic and anodic peaks. *E* ^0’^ was estimated as (*E*_p,c_ + E_p,a_)/2.

Figures 1 **b–** [Fig fig1] **e** show the CVs recorded at different *v* for the CNP immersed in an aqueous solution of 0.3 M KCl and 7.7 × 10^–4^ M K_4_Fe^[II]^(CN)_6_. The use of a supporting electrolyte at concentrations higher than 0.01 M is known to reduce the influence of the carbon surface charge on the electrochemical response.^24^ At low scan rates (<5 mV s^–1^), the voltammogram was characterized by a sigmoidal curve with a limiting current at bias voltages higher than 0.2 V. This response was ascribed to the electrochemical reaction of the redox probe onto the inlaid disk of the external CNP surface.^14^ At low scan rates, the contribution to the current from the reaction inside the CNP (thin-layer regime) is minimal; therefore, the electrochemical response was primarily determined (and limited) by the hemispherical diffusion of the redox probe in the external solution toward the outer carbon layer. This leads to CVs similar to those traditionally observed in ultramicroelectrodes.

The magnitude of the steady-state limiting current can be used to estimate the tip size of the CNPs by the following expression:^14^

$$i_{d}=4xnFDC_{bulk}r$$ 1

where *n*, *F*, *D*, *C*_bulk_, and *r* are the number of electrons involved in the redox reaction, the Faraday constant, the diffusion coefficient (*D* = 7.30 × 10^–6^ cm^2^ s^–1^ for ferrocyanide), the bulk concentration of the redox probe, and the CNP tip radius, respectively. Notably, *x* is a dimensionless parameter related to the ratio between the outer radius of the pipet tip (i.e., considering the insulating glass wall) and the tip aperture, which is typically estimated to be 1.1 for this kind of nanofluidic device.^14,25^ Since the limiting current showed values of 0.018 nA (at 0.4 V), the estimated *r* was ∼80 nm, comparable to the value obtained by SEM characterization (∼50 nm) (Figure S1). The slight differences between these two results may be attributed to sample-to-sample variations and the difficulty in precisely measuring the diffusion current, which is affected by a slight drift.

When *v* was increased from 5 mV s^–1^, a distinct peak pair emerged at ca. 0.2 V. This is ascribed to the significant contribution of the ferrocyanide ion redox reaction occurring within the CNPs to the overall electrochemical signal. The peak current associated with this internal redox reaction was found to increase with *v*. Then, at *v* higher than 50 mV s^–1^, the contribution of the inner walls of the CNP to the response fairly exceeded the diffusion-limiting steady-state current provided by the redox reaction at the CNP orifice. Moreover, the peak current (after capacitive current subtraction) for the cathodic (*i*_p,c_) and anodic (*i*_p,a_) peaks at the different *v* denoted a linear trend (*r* ^2^ > 0.999) from 5 to 250 mV s^–1^, as observed in Figure 1f. A comprehensive analysis based on numerical simulation of the interplay between the currents due to the internal CNP domain and the external carbon ring is available in Figure S3. Overall, the finding demonstrated that the CV time scale (at scan rates between 1 mV s^–1^–250 mV s^–1^) is sufficient for a complete redox probe consumption inside the pipet and in the proximity of the external carbon ring when *E* ≫ *E* ^0’^. Furthermore, for a given solution depth, the voltammetric response arises from both internal and external domains. Then, the total analyte consumption within the CNP, which is proper of thin-layer regimes, results in the bell-shaped pair of peaks where i_p_ increases linearly with *v*. On the other hand, the hemispherical concentration distribution near the carbon ring generates a diffusion-limited current, whose magnitude remains independent of v. These different dependencies with the scan rate explain why at low values the electrochemical response resembles that of traditional microelectrodes, whereas at high scan rates, it transitions to a thin-layer regime.

The voltammetric charge (*Q*), calculated as the ratio between the peak area of the CV and *v*, for the cathodic (*Q*_c_) and anodic (*Q*_a_) peaks displayed variations lower than 10% in terms of *v*, demonstrating the independence of *Q* with the perturbation velocity. The half-height peak was around 90 mV, which pointed out an ideal voltammetry behavior. In addition, the potential separation between the cathodic and anodic peaks (Δ*E*_p_) was under 15 mV at all *v* conditions (Figure 1g).^26^ All these trends are indicative of a thin-layer regime. Importantly, under these conditions, *Q* can be directly related to the redox probe moles (*N*_REDOX_) inside the nanopipette by the Faraday law.^14,27^ Accordingly, we used eqs 2 and ([Disp-formula eq3]) to estimate the sample volume (*V*) inside the CNP. A volume of 3.70 ± 0.06 pL was calculated from the CVs obtained at scan rates of 100, 150, 200, and 250 mV s^–1^.

$$Q=nFN_{REDOX}$$ 2

$$N_{REDOX}=C_{bulk}V$$ 3

### EIS Response at Different Direct Current Voltages

EIS measurements were conducted at three different *E*_DC_: 0 V, *E* ^0’^, and 0.4 V (Figure 2). This strategy aimed to evaluate the system at two voltages far from the formal potential of the redox probe, one below (0 V) and another one above (0.4 V), and at a third one where the reduced and oxidized forms of the redox probe coexist (*E* ^0’^). Before the EIS experiment, an *E* ^0’^ of 0.2 V was determined for the redox couple from the CV at 50 mV s^–1^ (details in Section 1 in the Supporting Information). Also, we confirmed that under ±10 mV of harmonic perturbation, the linear requirement of EIS measurements was still valid by comparing the impedance spectrum with those obtained using ±5 and ±20 mV (Figure S4). The quality of the EIS data was assessed by checking its consistency with the Kramers–Kronig relations (Figure S5).

**Figure 2:**
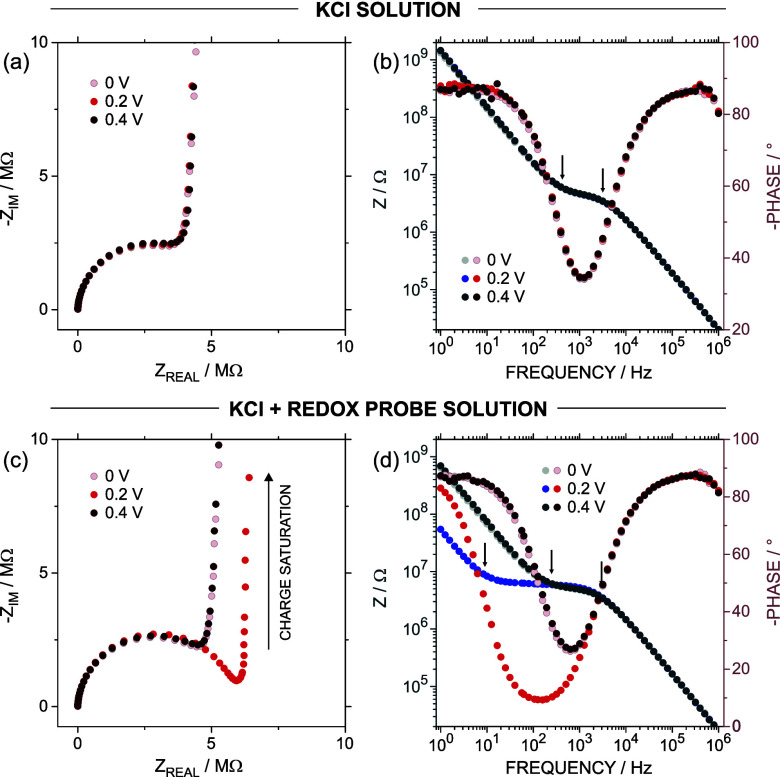
(a) Nyquist and (b) Bode plots at different E_DC_ for measurements conducted in a 0.3 M KCl solution. (c) Nyquist and (d) Bode plots at different E_DC_ for measurements performed in a solution containing 0.3 M KCl + 7.7 × 10^–4^ M Fe^(II)^(CN)_6_^4–^. The arrows in the Bode Plot indicate the breaking points.

Figures 2a and 2b present the EIS results at the three different *E*_DC_ for a CNP immersed in a solution containing 0.3 M KCl (i.e., experiments in the absence of a redox probe), while Figures 2c and 2d display the EIS results in a solution containing 0.3 M KCl and 7.7 × 10^–4^ M K_4_Fe^[II]^(CN)_6_ (i.e., experiments in the presence of a redox probe). In the absence of the redox probe, the Nyquist representations for the EIS spectra at *E*_DC_ = 0, 0.2, and 0.4 V showed very similar responses, characterized by a semicircle at high and medium frequencies, which is typical of a system with an equivalent circuit consisting of a resistance and a capacitance in parallel (RC) (Figure 2a, Nyquist plots in the complete frequency range are available in Figure S6). Specifically, the semicircle was characteristic of ion transport events in nanofluidic devices, with the resistive and capacitive contributions attributed to the tip resistance and the capacitance of the nanopipette due to the electrolyte distribution inside the pipet and around the surface, respectively.^1,21–23^ Therefore, the *Z*_REAL_ values at the beginning (high frequency) and end (medium/low frequency) of the semicircle (i.e., where *-Z*_IM_ tends to 0 Ω) correspond to the bulk solution resistance and the sum of the tip and bulk solution resistance (∼5 MΩ), respectively.^1^

The magnitude of the tip resistance was found to be highly sensitive to the size and geometry of the CNP. A higher radius and lower taper of the CNP resulted in a smaller semicircle in the Nyquist plot, indicating that nanofluidic devices with larger dimensions exhibit lower ion transport resistance (Figure S7).^21^ Importantly, this behavior revealed that slight variations during the nanofabrication of the CNP can drive to sample-to-sample variations in the EIS spectra. Therefore, we used the same CNP in each experimental case to ensure that we are able to evaluate the influence of certain variables of interest (e.g., redox probe concentration and volume). In addition, changes in the supporting electrolyte concentration and nature produced an analogous behavior. For instance, the increment in the KCl concentration produced a decrease in the semicircle diameter due to the decrease in the ion transport resistance. The replacement of potassium by tertbutyl ammonium, a cation of lower mobility, generated an increase in the semicircle, suggesting an increase in ion transport resistance. These results reinforce the hypothesis of a semicircle related to the ion transport process through the tip. A more detailed analysis is available in ESI (Figures S8 and S9).

At low frequencies, the Nyquist plot exhibited a sharp increase in *Z*_IM_ at the point of intersection with the *x*-axis, forming an angle close to 90°, which is indicative of a capacitive behavior due to the electrical double layer generated during the polarization of the carbon surface (Figure 2a). Then, it is crucial to emphasize that the *E*_DC_ magnitude (spanning from 0 to 0.4 V) does not substantially influence either ion transport or charging current, as demonstrated by the similarities in the EIS obtained at the various voltages in the absence of any redox probe. The EIS impedance in the presence of 7.7 × 10^–4^ M Fe^[II]^(CN)_6_^4–^ evidenced similar results at *E*_DC_ = 0 and 0.4 V to those obtained in the absence of the redox probe (Figure 2c). Figure S10 shows the overlapping of the EIS results in the presence and the absence of the redox probe at *E*_DC_ = 0 V, revealing an almost equivalent response. This result indicated that, at *E*_DC_ = 0 and 0.4 V, the response is solely dictated by the ion transport across the tip and the charging current on the carbon layer. This occurs because the harmonic voltage perturbation of 10 mV imparted during EIS experiments was too low to induce the oxidation of Fe^[II]^(CN)_6_^4–^ at *E*_DC_ = 0 V (or the reduction of Fe^[III]^(CN)_6_^3–^, for the case of the measurements at *E*_DC_ = 0.4 V), as these *E*_DC_ values are too far from *E* ^0’^.

The Nyquist plot for the EIS at *E*_DC_ = *E* ^0’^ = 0.2 V showed a semicircle very similar to those obtained at *E*_DC_ = 0 and 0.4 V but displaying some distortions in the medium/low-frequency range (Figure 2c). This difference is because part of the redox probe inside the CNPs is reduced (ferrocyanide form), while a significant portion remains oxidized (ferricyanide form). Under these conditions, the voltage variations caused by the harmonic perturbation are sufficient to trigger redox reactions of ferro/ferricyanide ions. Consequently, the EIS response is modulated by both ion transport through the CNP tip and the electron transfer of the redox probe on the carbon layer. Beyond such distortion of the semicircle, the redox contribution did not introduce any significant change in the Nyquist plot at high-medium frequencies. Finally, the system exhibited an almost vertical line at low frequencies, resembling the trends observed at *E*_DC_ = 0 and 0.4 V. This capacitive behavior at low frequencies is recognized as one of the EIS fingerprints in devices exhibiting thin-layer behavior, which also agrees with the trends observed in CV measurements.^26^ To our knowledge, this constitutes the first observation of such a trend reported for a nanofluidic device. The overall behavior has been rationalized in the ESI file (Figure S6).

Certain trends observed in the EIS data become more apparent when represented as Bode plots (both total impedance and phase angle as a function of frequency). For the EIS recorded at *E*_DC_ = 0 and 0.4 V, either in the absence or presence of the redox probe, the phase angle module presented values close to 80–90° at high and low-frequency values, indicating a capacitive behavior (Figure 2b and 2 **d**). At frequency values around 1 kHz, the phase angle showed its minimum magnitude (20°), coinciding with a frequency-independent *Z* region with an approximated magnitude of 5 MHz (representing the pore resistance). At low frequencies (<100 Hz), the phase angle approached again 90°, denoting the pure capacitive behavior of the charge saturation region. The Bode plot obtained at *E*_DC_ = *E* ^0’^ displayed a similar trend but with a decrease in the phase angle minimum from 20° to 9° and a broadening toward lower frequency values. Additionally, the frequency range where Z remains frequency-independent was extended, shifting the frequency at which the charge saturation occurs to lower values.

One approach to shed light on the different time scales involved in each process is through the analysis of frequency breaking points in the Bode plots.^18,28^ These breaking points are identified as the regions where the curve *|Z|* vs frequency evidence changes in the slope (which generally aligns with the frequency at which the phase angle is 45°). As shown in Figures 2b and 2 **d**, a breaking point at high frequencies indicates the transition of *|Z|* from a capacitive to resistive behavior, while another breaking point at moderate-low frequencies marks the transition from resistive to capacitive behavior (both highlighted by black arrows). The high-frequency breaking point, located at 2600 Hz (3.85 × 10^–4^ s), remained consistent across the different *E*_DC_ values, as it depends on the capacitance of the nanofluidic device and the tip resistance, both of which are independent of the applied voltage. However, in the presence of the redox probe (Figure 2d), the low-frequency breaking point–associated with the transition from tip resistance to charge saturation capacitance–was found to vary. For *E*_DC_ = 0 and 0.4 V, this breaking point occurred at the same frequency of 160 Hz (6.25 × 10^–3^ s), whereas at *E*_DC_ = *E* ^0’^, it shifted to 9 Hz (0.11 s). This frequency shift can be explained by considering the physicochemical processes underlying the system’s low-frequency capacitive behavior. At *E*_DC_ = 0 and 0.4 V, the capacitance at low frequency arises primarily from the charging of the electrical double layer; whereas at *E*_DC_ = *E* ^0’^, it involves both the charging of the electrical double layer and the accumulated charge due to the electron transfer of the redox probe, explaining the observed shift in the frequency.

Overall, the results in Figure 2 suggested various key insights. First, the response is mainly given by the ion transport across the CNP for EIS recorded at an *E*_DC_ far from *E* ^0’^. Second, when the applied *E*_DC_ is close to *E* ^0’^, electron transfer seems to be coupled with the ion transport across the tip. Similarities in the EIS spectra at different *E*_DC_ values demonstrate that the ion transport contribution primarily determines the semicircle at high-moderated frequencies. Third, when *E*_DC_ ∼ *E* ^0’^, the capacitive behavior associated with charge saturation shifted to lower frequencies compared to the situation revealed at *E*_DC_ = 0 V. This shift is due to the contribution of the redox reaction, which requires more time to occur (lower characteristic frequency) compared to the charging of the carbon surface. Thus, analyzing the EIS response at different *E*_DC_ values in the presence of the redox probe allowed for the differentiation between faradaic, ion transport, and capacitive contributions, showcasing one of the key strengths of this technique when studying complex electrochemical systems. A more comprehensive analysis of the fundamentals of EIS related to the thin-layer domain is available in Figure S6. Moreover, as demonstrated in Figure S11 and Figure S12, it is also possible to extract quantitative information from each process by performing a fitting based on electrical equivalent circuits.

### Electrochemical Response at Different Concentrations of the Redox Probe

Figure 3 shows CV and EIS experiments at varying redox probe concentrations. In the CVs, the peak potentials (*E* ^0’^ = 0.2 V) did not show any significant change as either *v* or the redox analyte concentration were varied (see Figure S13 for the raw data). For instance, Δ*E*_p_ for the voltammograms of 0.75 mM Fe^[II]^(CN)_6_^4–^ and scan rates of 50 mV s^–1^ and 250 mV s^–1^ were 5 mV and 11 mV, respectively. An increment of the concentration of the redox probe to 2.5 mM led to Δ*E*_p_ of only 10 mV and 21 mV for 50 and 250 mV s^–1^. It is here anticipated that only slight changes in the peak potentials with the scan rate and ferrocyanide concentration were observed, which can be attributed to the less influence of ion resistance on the CVs when the experiments are performed in CNP with a low filling degree (see below). In contrast, for CNPs with higher solution depths, it is expected an accentuation in Δ*E*_p_ when the redox probe concentration is increased, due to limitations given by the ion transport. This behavior was further confirmed and analyzed by numerical simulations (Figure S14).

**Figure 3:**
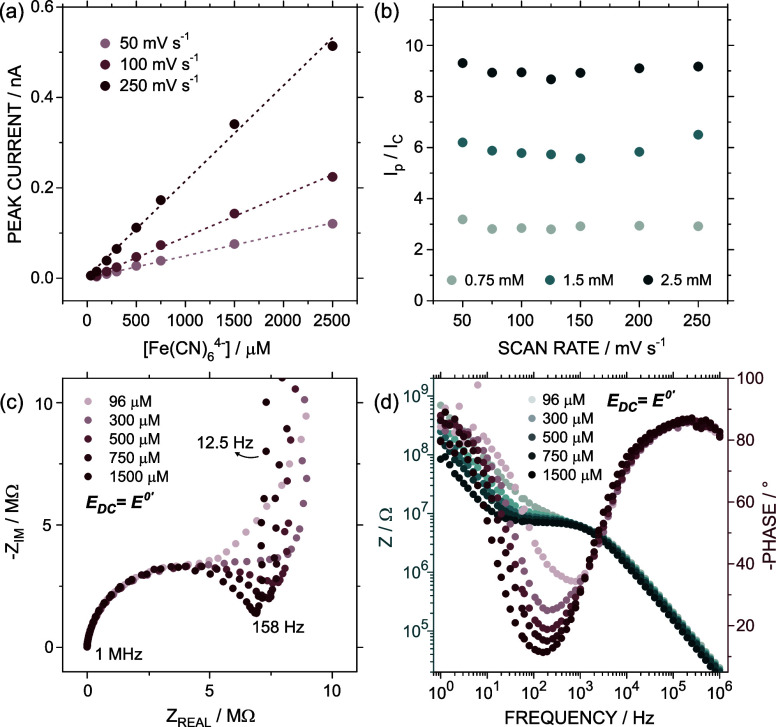
(a) Plot of *I*_p_ vs the used ferrocyanide concentration ([Fe^[II]^(CN)_6_^4–^]) for *v* = 50, 100, and 250 mV s^–1^. (b) Ratio of the peak and capacitive currents (*I*_p_/*I*_c_) at increasing v from CVs obtained in [Fe^[II]^(CN)_6_^4–^] = 750 μM, 1500 μM, and 2500 μM. (c) Nyquist and (d) Bode plots obtained at *E*_DC_ = *E* ^0’^ for different [Fe^[II]^(CN)_6_^4–^]. Supporting electrolyte: 0.3 M KCl.

The increases in the redox probe concentration are translated into a linear increase in the peak current in the voltammograms. Moreover, for a given analyte concentration, higher *v* increased both the current magnitude and calibration sensitivity (slope) (Figure 3a). Note that the slope (in units of nA/mM) increased from 0.0487 ± 0.0009 to 0.092 ± 0.002 and 0.211 ± 0.006 when *v* was increased from 50 mV s^–1^ to 100 and to 250 mV s^–1^, respectively. This trend is typically obtained in devices exhibiting thin-layer behavior as opposed to macroscopic electrodes. Indeed, in macroscopic systems, increasing *v* does not necessarily enhance the calibration sensitivity: while the peak current must increase with *v* ^1^/,^2^ *I*_c_ must do it linearly with *v*, causing the *I*_p_/*I*_c_ ratio to decrease.^26^ In the nanoscale system, *I*_p_ varies linearly with *v*, maintaining a constant*I*_p_/*I*_c_ ratio through the different *v* (Figure 3b). Despite the increment in *v* representing a favorable scenario in terms of current magnitude, the use of higher *v* (especially for low current recording) requires instruments with enhanced bandwidth capabilities.

The comparison of the EIS results at *E*_DC_= 0 V for different redox probe concentrations showed no significant variations in both Nyquist and Bode plots. This can be attributed to the fact that the addition of the redox probe in concentrations <2500 μM does not generate appreciable changes in the ionic strength fixed by the supporting electrolyte (0.3 M KCl) and, consequently, in the pore resistance (see Figure S15). Importantly, this fact reinforces the hypothesis that the signal is mainly determined by the ion transport contribution at *E*_DC_ = 0 V. Conversely, major changes were obtained for the measurements at *E*_DC_ = *E* ^0’^ (data in a wider frequency range is available in Figure S15). As the redox probe concentration increased, the distortion in the semicircle in the Nyquist plot diminished, likely due to a reduced contribution of the charge-transfer resistance to the overall behavior (Figure 3c). In fact, at high redox probe concentrations, the Nyquist plot resembled those obtained at *E*_DC_ = 0 V but showed an almost complete semicircle.

In the Bode plot, unlike the 0 V case, the increment in the redox probe concentration resulted in significant changes in the phase angle (Figure 3d). Specifically, higher concentrations led to a decrease and a broadening of the minimum phase angle. For instance, the phase angle minimum decreases from 28° to 16° when the concentration is increased from 300 μM to 1500 μM. Thus, as the concentration increased, the frequency at which the charge saturation occurs shifted toward lower values. In this regard, the analysis of the breaking points showed frequencies for the transition from resistive to capacitive (charge saturation) behavior of 120, 32, and 13.5 Hz for concentrations of 96, 750, and 1500 μM.

### Impact of the Sample Volume on the Electrochemical Performance

Figure 4a displays successive cycles of CVs obtained in a CNP immersed in a solution of 0.3 M KCl and 7.7 × 10^–4^ M K_4_Fe^[II]^(CN)_6_ before volume stabilization. By integrating the voltammetric peak, the volume of the CNPs was estimated by applying the Faraday law. Figure S16 replicates the experiment in Figure 4a with a twin sample and includes optical microscopy images captured at different solution depths to demonstrate the volume variations. It was found that the increment in the volume inside the CNP generated marked variations in *I*_c_, *I*_p,_ and *E*_p_ for both oxidation and reduction reactions (Figure 4b–d). Also, such variations are accentuated by increasing the scan rate (a detailed analysis is available in Figure S17).

**Figure 4:**
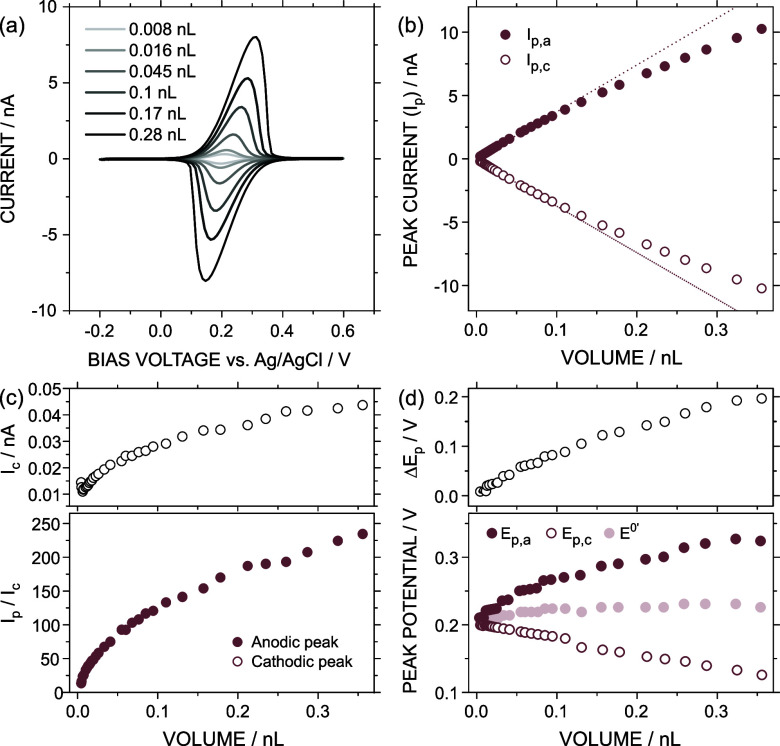
(a) CVs obtained for the same CNP at increasing filling volumes. (b) Plot of I_p_ vs the volume inside the CNP. (c) Plots of I_c_ (top) and I_p_/I_c_ ratio (bottom) versus the volume inside the CNP. (d) Plots of the peak potential separation (top) and peak potentials (bottom) versus the volume inside the CNP. All the measurements were conducted in 0.3 M KCl + 7.7 × 10^–4^ M Fe^[II]^(CN)_6_^4–^.

The peak currents obtained for the cathodic and anodic reactions evidenced a monotonic increase as the pipet volume increases, with a slight deviation from the linear trend at volumes > ∼ 100 pL (Figure 4b). For instance, the anodic peak current increased from 0.31 nA to 1.6 nA when the volume increased from 8 pL to 40 pL. This trend was attributed to the higher number of redox-active moles inside the CNPs due to the rise in volume. The deviation from the linear trend at higher volumes is likely due to the loss of the typical Gaussian peak shape (peak distortion) under the thin-layer regime, resulting from the uncompensated ion resistance at the tip. Similarly, *I*_c_ was found to increase as the solution depth grew (Figure 4c). The *I*_p_/*I*_c_ ratio rapidly increased with the pipet volume when the solution depth was still low, and then, the rising velocity was attenuated, although the solution depth maintained its growth. For instance, the increment of the volume from 8 pL to 40 pL produced an enhancement of the *I*_p_/*I*_c_ of ∼ 3 times. Such magnitude reached ∼ 10 times if the volume increases from 5 pL to 110 pL. This finding constitutes a valuable insight into the use of CNP devices for sensing applications. Increasing the volume not only creates favorable conditions in terms of the *I*_p_/*I*_c_ ratio but also results in higher current values, which is advantageous for devices of this kind, where the signal magnitude may approach the limit of the potentiostat. Also, the thin-layer properties are maintained in all the cases, which would enable calibration-free coulometric determinations.

To further test the hypothesis that higher volumes can be an advantage due to the maximization of the *I*_p_ */I*_c_ ratio, CVs were continuously recorded on a CNP immersed in a highly diluted solution of 7.7 × 10^–7^ M K_4_Fe^[II]^(CN)_6_ in 0.3 M KCl until the volume stabilized (Figure S18). Initially, no peaks were observed due to the low solution volume and, consequently, the insufficient number of analyte moles inside the CNP to generate a detectable faradaic signal. Then, as the number of CV cycles increased, capillary rise caused the internal solution volume to advance inside the nanofluidic device. This was reflected in a noticeable increase in capacitive current, attributed to the growing electrochemically active area as the solution depth increased. After 80 cycles, the volume stabilized, and the faradaic signal due to the presence of the redox probe became constant and quantifiable. Furthermore, as discussed above, this faradaic signal can be amplified if the CV is conducted at higher *v* (Figure S19).

Truly, the peak potential was found to be very sensitive to the solution depth. For example, pipet volumes of less than 0.025 nL resulted in Δ*E*_p_ below 20 mV. Such a value, appreciably lower than 59 mV, is characteristic of electrochemical reactions under thin-layer regimes (ideally, it should tend to 0 mV). However, the increment in the volume rapidly led to significant peak potential separations; for instance, 150 mV for volumes around 0.5 nL. As a result, the peak potential separation rapidly deviated from the typical thin-layer behavior. These trends were rationalized by employing numerical simulations (Figure S20).

To gain more insight into the different phenomena occurring during the electrochemical response of CNPs with varying volumes, we conducted EIS at *E*_DC_ = 0 V and *E* ^0’^ in 0.3 M KCl + 7.7 × 10^–4^ M K_4_Fe^[II]^(CN)_6_. The results are depicted in Figure 5 (complete spectra available in Figure S21). The Nyquist plot does not seem to show significant changes in terms of resistance since, in all the cases, the response is characterized by a semicircle of similar magnitudes (Figure 5a). This trend can be attributed to the conical geometry of the CNPs.^29^ In conical geometries, the total ion resistance is primarily concentrated in the tapered shaft near the tip. Consequently, increasing the liquid column beyond a certain depth does not significantly affect the ion resistance.

**Figure 5:**
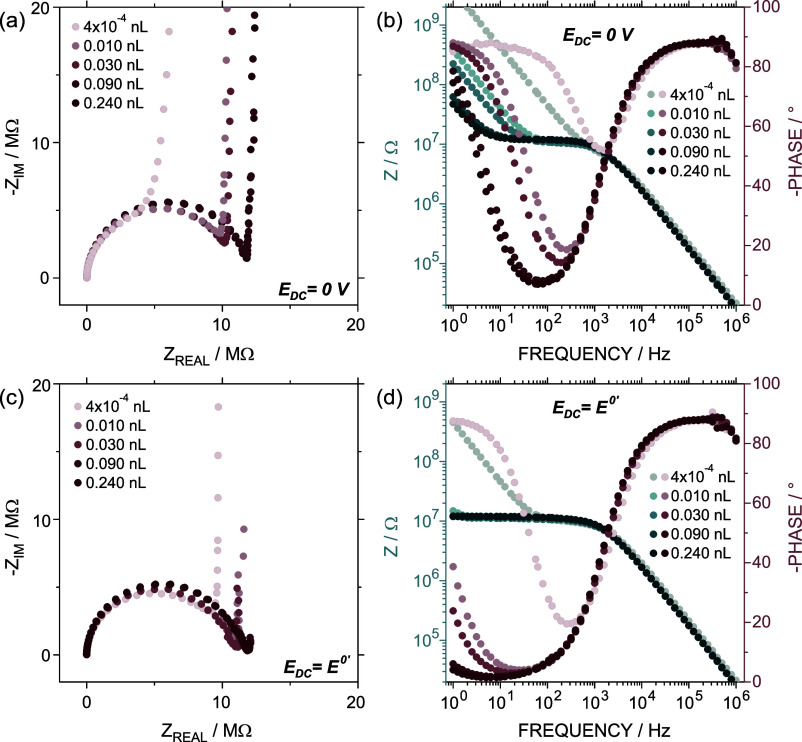
(a) Nyquist and (b) Bode plots for a CNP with different solution volumes at *E*_DC_ = 0 V. (c) Nyquist and (d) Bode plots for a CNP with different solution volumes at *E*_DC_ = *E* ^0’^ = 0.2 V. All the measurements were performed in a mixture of 0.3 M KCl + 7.7 × 10^–4^ M Fe­[II]­(CN)_6_^4–^.

An interesting aspect is that, as the internal volume increases, the frequency at which the charge saturation happens due to double-layer charging shifts to lower values. This fact is more evident in the Bode plot (Figure 5b), in which the frequency region where the impedance behaves independently of the frequency is widened, and the phase angle minimum becomes broader and takes lower values as solution depth increases (from 50° to ∼10°). The shift of this region toward lower values indicated an increase in the double-layer capacitance, which agrees with the increment of the capacitive current in terms of volume. Regarding the breaking points, this fact is transduced into a decrease in the frequency of the location of the low-frequency breakpoint. More in detail, the frequency took values of 40, 21.55, and 3.72 Hz for the EIS performed with volumes of 0.01, 0.03, and 0.24 nL, respectively.

Similarly, at *E*_DC_ = *E* ^0’^, there were no significant changes in the semicircle when different filling levels were compared (Figure 5c). Moreover, the asymptotic growth of the–*Z*_IM_ at low frequencies shifted toward lower frequencies as the filling degree increased. The analysis of the Bode plot follows a similar trend to the previous case: the phase angle minimum broadens and decreases in absolute value (from 20° to ∼ 0°) with increasing filling levels (Figure 5d). Under this condition, the minimum reaches lower phase angle values and is wider compared to the *E*_DC_ = 0 V case, which is likely due to the contribution of the redox reaction (e.g., for the maximum volume analyzed, 10° vs ∼0° at 0 V and *E* ^0’^, respectively). Therefore, at both *E*_DC_ conditions, a shift in the frequency of the low-frequency vertical line toward lower values is produced because of the increment of the charge saturation capacitance. In the case of *E*_DC_ = *E* ^0’^, the shift causes the low-frequency breakpoint to no longer be observed within the evaluated frequency range. This suggests that the time required to completely oxidize or reduce all the analyte moles inside the CNP (exhaustive behavior) is delayed as the filling degree increases.

## Conclusions

The study provides a comprehensive analysis of the electrochemical behavior of CNPs, with a focus on the thin layer properties. This was evidenced in CV experiments at different conditions by the linear trend of peak currents with *v*, constant voltammetric charge, and the minimal potential separation between peaks. However, large sample volumes (>100 pL) led to deviations from the well-known fingerprints of the thin-layer behavior. Furthermore, an enhancement of the signal-to-background ratio of more than 50 times was revealed, which is undoubtedly beneficial for future sensing applications. In EIS measurements at different *E*_DC_, the coupling of ion transport and electronic transfer has been demonstrated. Overall, the EIS response primarily reflects the ion transport across the CNP tip; however, close to the formal potential of the redox couple used in our experiments (Fe^II/III^), both ion transport and electronic transfer sensitively influence the response. EIS analysis allowed us to deconvolute these two contributions by modulating the *E*_DC_, enabling a deeper insight into their roles in the electrochemical response. Importantly, observations herein highlighted the intricate balance between ion transport and electronic transfer in nanoscale electrochemical systems, emphasizing the need for a deeper understanding of these processes to optimize CNP-based devices for sensing and other applications, which sometimes remain undervalued. Moreover, given the distinct information provided by each type of signal in this kind of device, the dual-mode functionality of CNPs holds considerable promise for advanced sensing applications and broader electrochemical investigations, including electrocatalysis and imaging.

## Supplementary Material



## Notes

Materials methods; microscopy analysis; additional EIS at different experimental conditions; additional CVs at different experimental conditions; COMSOL model details (PDF)
