# Supplementary material for: Thin-Layer Behavior in Carbon Nanopipettes. Understanding the Iontronic-Electronic Contributions
Source: Anal Chem. 2025 Aug 5;97(32):17659–67. doi: 10.1021/acs.analchem.5c02834 (PMC12368838; doi:10.1021/acs.analchem.5c02834)
Supplement: Supplementary file 1 [file ac5c02834_si_001.pdf]

Supporting Information for:

Thin-Layer Behavior in Carbon Nanopipettes. Understanding the Iontronic-Electronic Contributions

Gregorio Laucirica[a], Gastón A. Crespo[a,b], María Cuartero[a,b,*]

[a]UCAM-SENS, Universidad Católica San Antonio de Murcia, UCAM HiTech, Avda. Andres Hernandez Ros 1, 30107, Murcia, Spain.

[b]Department of Chemistry, School of Engineering Science in Chemistry, Biochemistry and Health, KTH Royal Institute of Technology, Teknikringen 30, SE-114 28 Stockholm, Sweden.

Table of contents

1. Materials and Methods .... Error! Bookmark not defined.

2. Microscopy analysis **6**

3. Additional figures **7**

Contribution of internal and external domains 7

Linearity test 9

Kramers-Kronig validity test 10

EIS in the absence and presence of redox probe 11

EIS of different CNP geometries 12

Electrochemical response at different supporting electrolyte concentrations 13

EIS response in the presence and absence of redox probe 15

Equivalent circuit analysis. 16

Cyclic voltammetries and EIS at different redox probe concentrations 19

Electrochemical response at different inner volumes. 21

4. Additional tables **25**

5. References **26**

Annex 1. Protocol for numerical simulations (COMSOL report) **28**

1. Definitions **29**

1.1 Parameters 29

1.2 Shared Properties 29

1.3 Model entry 29

2 Component 1 .... **30**

2.1 Definitions 30

2.2 Coordinate Systems 30

2.3 Geometry 30

2.4 Materials 30

2.4.1 KCl 0.3 M *31*

2.4.2 Graphite 1 .... *32*

2.5 Electroanalysis 32

2.5.1 Interface Settings *33*

2.5.2 Species Charges 1 .... *34*

2.5.3 Electrolyte *35*

2.5.4 Axial symmetry *38*

2.5.5 No flux *38*

2.5.6 Isolation 1 .... *39*

2.5.7 Initial values 1 .... *40*

2.5.8 Electrolyte potential 1 .... *40*

2.5.9 Concentration 1 .... *41*

2.5.10 Electrode surface 1 .... *42*

2.6 Mesh 48

2.6.1 Size *48*

2.6.2 Free Triangular 1 (ftri1) *48*

3 Study 1 .... **50**
3.1 Parametric Sweep 50
3.2 Cyclic Voltammetry 50
3.3 Solver Configurations 50
3.3.1 Solution 1 .... *50*

1. Experimental Section

Materials. Potassium chloride (99.5%, KCl), ferrocene methanol (97%), and $K_4Fe^{[II]}(CN)_6 \cdot 3H_2O$ (99%) were purchased in VWR chemicals, while tetrabutylammonium chloride (≥97.0%, TBACl), Ag wires (0.5 mm diameter) and sodium hypochlorite solution (6–14% chlorine) were provided by Merck. Quartz capillary tubes without filament (0.7 mm inner diameter, 1.0 outer diameter, and 10 cm length) were obtained from Sutter Instrument (Novato, CA). All the reagents were employed without any further treatment. All solutions were prepared in ultrapure water (18.2 MΩ cm at 25°C, Milli-Q® water system, Merck Millipore).

Glass nanopipette fabrication. Two kinds of nanopipettes were fabricated from quartz capillaries by employing a CO_2 laser-based puller P-2000 (Sutter Instrument). The programs employed for the fabrication are detailed in **Table S1**: Program 1 and Program 2. Analysis by scanning electron microscopy (SEM, Apreo S, Thermo Fisher Scientific) showed tip radii around 50 nm and 500 nm for the CNPs obtained with programs 1 and 2, respectively (**Figure S1**). Nanopipettes fabricated by Program 1 were used in the experiments unless otherwise indicated.

Table S1. Pipette pulling parameters employed in this work.

Program number	Heat	Filament	Velocity	Delay	Pull
1	700	4	60	145	175
2	700	4	30	130	90

Fabrication of carbon-coated glass nanopipettes (CNPs). A carbon layer was deposited onto the inner surface of the glass nanopipettes by chemical vapor deposition (CVD).[1,2] For this, the glass nanopipettes were exposed to a mixture of CH_4:Ar 0.2:0.6 L min^{-1} for 3.5 minutes at 925 °C. In all the cases, the tip side of the CNP faced the entrance of the gas line. The carbon layer on the inner surface imparts the electrical properties needed for using the CNP as a working electrode (WE).

Electrochemical setup. All electrochemical experiments were performed using a three-electrode setup consisting of a Pt rod (1.92 mm diameter, electroactive area > 2 cm^2, Metrohm Nordic AB, Sweden) as the counter electrode (CE) and a homemade Ag/AgCl wire as the reference electrode (RE). The Ag/AgCl reference electrode was prepared by immersing an Ag wire (0.5 mm diameter, 5 cm of length) into a diluted sodium hypochlorite solution (0.6–1.4% chlorine active) for 2 hours. The inner surface of the CNP was used as the working electrode (WE). For this, an Ag wire was inserted into the back of the capillary (0.5 mm diameter, 7 cm length) to create the necessary electrical connection between the nanotip and the potentiostat socket. The dimensions of the Ag wire allowed it to remain securely fixed inside the capillary, ensuring good contact with the carbon surface without the need for any adhesive, ink, or glue. The electrodes (WE, CE, and RE) were positioned equidistant from each other, with a distance of approximately 1 cm, inside the measurement reservoir.

The electrodes were connected to a potentiostat VIONIC (Metrohm) operated with the Intello 1.5 software. The analyses (EIS fitting and Kramers-Kronig analysis) were conducted with the NOVA 2.1.4 software. All the experiments were carried out inside a Faraday cage (Rittal, GmbH & Co. KG).

CVs were typically recorded at 50 mV s^{-1} in the potential window between –0.2 V and 0.6 V, otherwise mentioned. Peak currents I_p were obtained by first subtracting the background (**Figure S2**). Capacitive currents I_c were determined from the flat region of the voltammograms. The reported values of I_c correspond to the average (absolute) obtained from the forward and backward scans. The volume inside the CNP was estimated by integrating the voltammetric charge of the peak.

EIS experiments were performed varying the frequency from 1 Hz to $1x10^6$ Hz, recording ten points per decade and applying a sinusoidal perturbation with an amplitude of ±10 mV, otherwise mentioned. The sinusoidal perturbation was superimposed on a given direct current potential (E_{DC}). Typically, the E_{DC} values were chosen as 0 V, formal potential ($E^{0'}$), and 0.4 V to investigate the system behavior under conditions that ensure both the presence and absence of redox reactions. Before redording the EIS, $E^{0'}$ is estimated from the CV response at 50 mV s^{-1} as $E^{0'} \sim (E_{p,c} + E_{p,a})/2$ (where $E_{p,c}$ and $E_{p,a}$ correspond to the cathodic and anodic peak potentials). Regarding this, small changes around the formal potential (±5–10 mV) do not significantly change the EIS response. In all the cases, the system was preconditioned at the E_{DC} for 10 seconds before the measurement.

For the equivalent circuit fitting, NOVA software was employed. Considering the relatively high supporting electrolyte concentration and the short distance between the WE and RE in the experimental setup (∼1 cm), the ohmic drop due to solution resistance R_{sol} was negligible compared to that of the nanofluidic device. The initial value of R_{sol} was extracted from the high-frequency intercept (in x-axis) and then fitted for MODEL 1 – 0 V. The obtained value was fixed for the rest of the tests because any change was performed in the electrochemical cell. However, it is worth mentioning that variations in this parameter between 0.5 and 6 kΩ do not generate substantial variations in the rest of the magnitudes obtained with the fittings (< 10%). This is because the contribution of R_{SOL} to the total impedance of the system is negligible (R_{sol} was around 1000 times lower than the tip ion resistance).

COMSOL simulations. Simulations of CV experiments were performed with *COMSOL Multiphysics v.6.1* with a 2D model with axial symmetry was employed. The electrochemical problem was solved with a model based on the "Electrochemistry" branch, with "Electroanalysis" as the physics interface. In essence, the model considers a Nernstian one-electron reaction occurring on a carbon surface with a kinetic reaction and the transport of species following the Butler-Volmer equation and Fick law, respectively. The charge conservation type was assigned to the supporting electrolyte to account for the effect of the ion transport through the tip. All the details of the model and calculations are available in the model report at the end of this document (**Annex**). Parameters including, tip radius, supporting electrolyte concentration, redox probe concentration, and scan rate were fixed following the values obtained (or fixed) experimentally. Notably, the cone angle (θ) and capacitance of the carbon layer were selected as tan(θ)=0.2 and 20 μF/cm^2, respectively, as reported elsewhere.[3–5]

2. Microscopy analysis

Geometric parameters of the carbon nanopipettes were analyzed by optical and scanning electron microscopies (SEM) (**Figure S1**). SEM characterization was conducted using a field-emission SEM (Apreo 2, Thermo Fisher Scientific®). For this purpose, the carbon nanopipettes (CNPs) were shortened to 1.5 cm, oriented at 90°, and coated with a 4 nm platinum (Pt) thin film via sputtering. Optical microscopy images were acquired with an inverted optical microscope (Nikon Eclipse Ti2) coupled to a DS-Qi2 camera from Nikon Instrument, Inc.

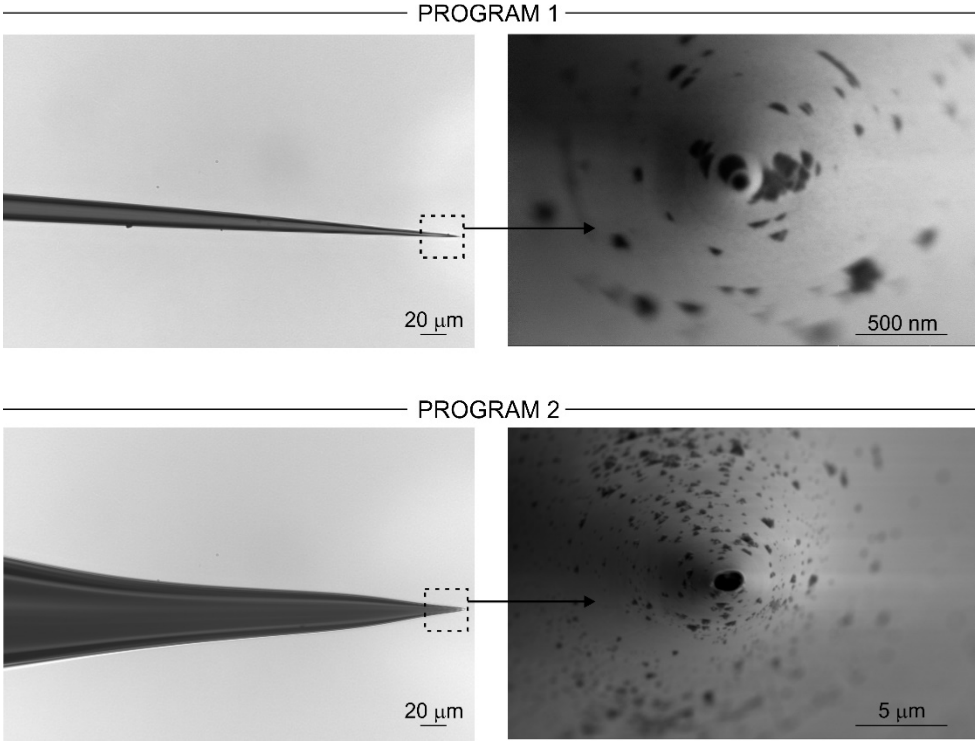

Figure S1. Microscopy characterization of CNPs fabricated by employing two different programs. The left panel figures correspond to optical microscopy images (40x), while the right panel figures correspond to scanning electron microscopy images (50k and 5k of magnification for programs 1 and 2, respectively).

3. Additional figures

Background subtraction

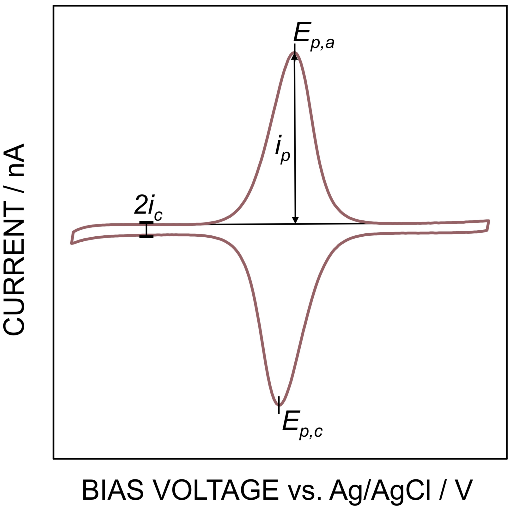

Figure S2. Scheme of the procedure used to extract the peak current (i_p), peak potentials (E_p), and capacitive currents from a voltammogram.

Contribution of internal and external domains

Simulations of the experimental CV calculations were conducted to investigate the contribution of the two distinct domains to the electrochemical response: the external carbon ring and the inner volume of the cone (**Figure S3a**, see ESI for model details). The overlapping experimental and calculated CVs at 1 mV s^{-1} and 50 mV s^{-1} are presented in **Figure S3b**, together with an illustration of the dominant CNP domains identified at each potential window.

Initially, at a bias voltage (*E*) of –0.2 V —below the formal potential $E^{0'}$ of the redox probe—, the ferrocyanide concentration remains uniform at 7.7 x 10^{-4} M (C_{bulk} value employed in the simulation) throughout the system due to the absence of any redox reaction (**Figure S3c**). As a result, the current values in the voltammograms when $E << E^{0'}$ exhibited only a capacitive contribution. Then, as *E* approaches $E^{0'}$ (E=0.2 V), the voltage perturbation becomes sufficient to trigger the redox reaction, altering the concentration distribution relative to the initial bulk value (**Figure S3d**). Inside the CNP, ferrocyanide concentration decreases by 50%, while in the reservoir (external domain), partial depletion occurs near the carbon ring and dissipates rapidly with increasing distance from the pipette. Finally, at sufficiently high voltages ($E >> E^0$), ferrocyanide is entirely consumed within the CNP (**Figure S3e**), while in the reservoir, the depletion near the carbon ring is accentuated with a hemispherical concentration distribution profile (inset in **Figure S3e**).

Overall, this finding demonstrates that the CV timescale (at scan rates between 1 mV s^{-1} – 250 mV s^{-1}) is sufficient for a complete redox probe consumption inside the pipette and in the proximity of the external carbon ring when $E >> E^{0'}$. Furthermore, for a given solution depth, the voltammetric response arises from both internal and external domains. Then, the total analyte consumption within the CNP, which is proper of thin-layer regimes, results in the bell-shaped pair of peaks where i_p increases linearly with *v*. On the other hand, the hemispherical concentration distribution near the carbon ring generates a diffusion-limited current, whose magnitude remains independent of *v*. These different dependencies with the scan rate explain why at low values the electrochemical response resembles that of traditional microelectrodes, whereas at high scan rates, it transitions to a thin-layer regime. This change in response due to the CNP geometry has been reported in previous studies.[3,6]

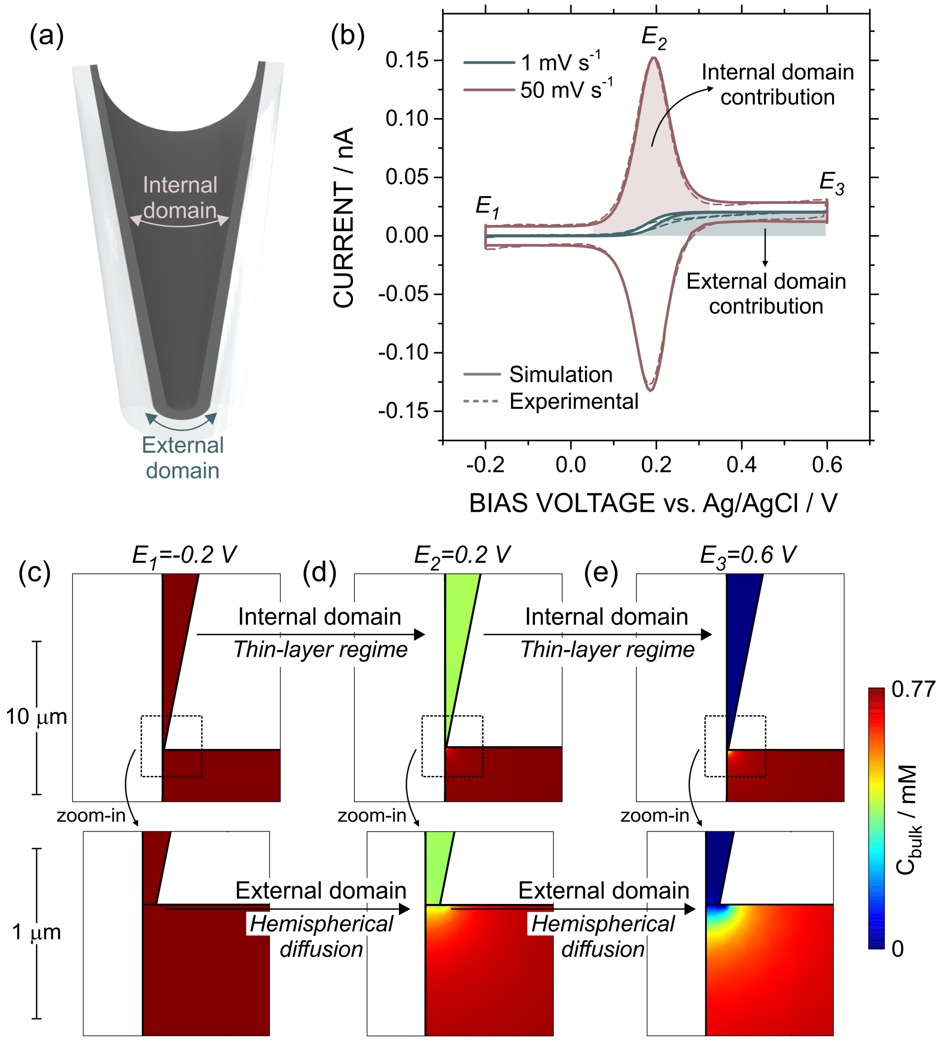

Figure S3. (a) Internal and external CNP domain scheme. (b) Experimental (dashed line) and simulated (full line) CVs at 1 mV s^{-1} and 50 mV s^{-1}. (c–e) concentration distribution of ferrocyanide at three different bias voltages during the CV experiments: –0.2 V, 0.2 V, and 0.6 V. Sample solution: 0.3 M KCl and 7.7 x 10^{-4} M $K_4Fe^{[II]}(CN)_6$. Tip radius 80 nm, cone angle: tan(θ)=0.2, double-layer capacitance: 20 μF/cm^2, and solution depth: 45 μm.

Linearity test

EIS experiments were performed by employing 10 mV as the amplitude of the harmonic perturbation. We confirmed that under such harmonic perturbation, the linear requirement of EIS measurements was still valid by comparing the impedance spectrum with those obtained using 5 mV and 20 mV (**Figure S4**). The results at 5 mV, 10 mV, and 20 mV were invariable, demonstrating that the system linearity was achieved under the three conditions. Notably, amplitudes of 10 mV were selected because, in comparison to 5 mV perturbations, they enable the maximization of the signal-to-noise ratio, and, at the same time, it was small enough to ensure the system's linearity.[7] Then, compared with 20 mV, there was not much improvement of the signal; but it was preferred to use 10 mV instead of 20 mV because it is a more standard value for EIS experiments and with a higher security of being within the linear regime.

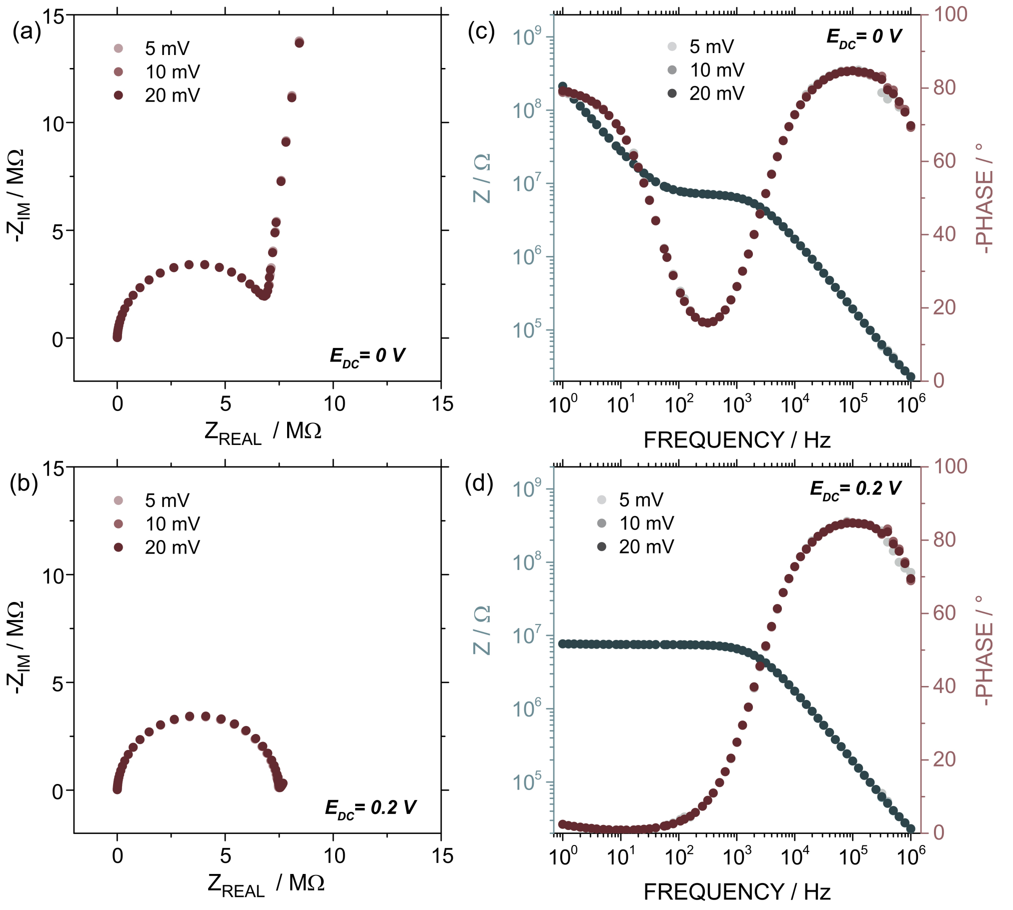

Figure S4. Nyquist and Bode plots for EIS experiments carried out at (a)(b) E_{DC}= 0 V and (c)(d) E_{DC}= 0.2 V at three different harmonic perturbation amplitudes. The plots are almost perfectly overlapped which demonstrates the low effect of the amplitude in the evaluated range. In all the cases the measurements were performed in a solution of 0.3 M KCl and with 0.77 mM of $K_4Fe(CN)_6$.

Kramers-Kronig validity test

Kramers-Kronig validity test demonstrated percentual errors lower than ±2% in both the real and imaginary Z components (Z_{REAL} and Z_{IM}) in the range of frequencies between 10 and 10^5 Hz (**Figure S5**). Typically, in the ranges of 1–10 and 10^5–10^6 Hz, the percentual error is found to increase, especially in the real component, owing to some instrumental limitations at such high impedance values.

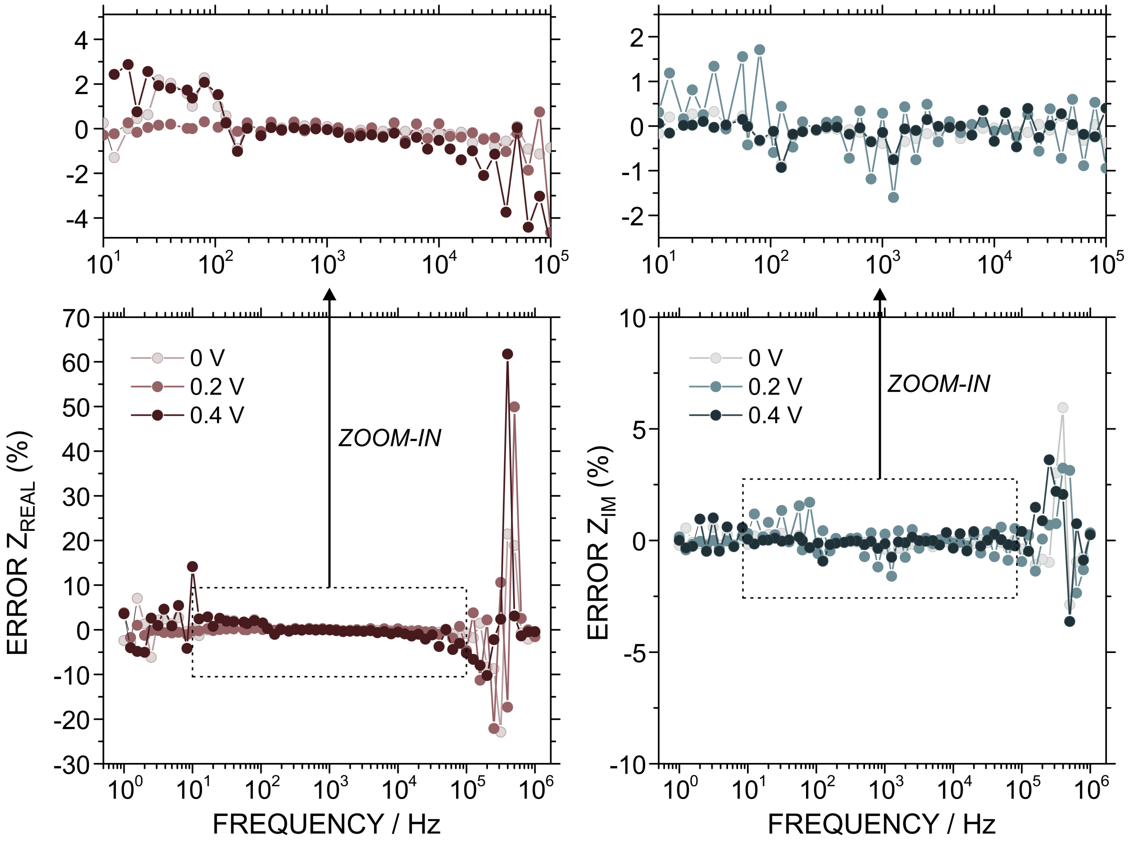

Figure S5. Error in the real and imaginary components of Z derived from Krammers-Kromig analysis.

EIS in the absence and presence of redox probe

EIS in the absence and presence of redox probes is characterized by a semicircle at high/moderate frequencies. This feature, also observed in iontronic experiments, is attributed to the solution capacitance inside the nanofluidic device and the tip resistance. This trend is very similar to those observed in EIS experiments conducted with macroelectrodes in the presence of a redox probe. However, in contrast to this kind of electrochemical experiments under a semi-infinite diffusion regime, the sudden increase in Z_{IM} at low frequencies observed in these nanoelectrodes differed significantly from the typical Warburg slope of ~1.[8,9] This characteristic behavior, generally observed in electrochemical experiments conducted either with dissolved redox probes under a thin-layer regime or when they are adsorbed onto the electrode surface, was rationalized as follows. At high-medium frequencies, the velocity of variations of the sinusoidal perturbation exceeded the velocity of the charge propagation in the solution, and therefore, the variations are only produced near the electrode surface. In other words, only the portion of the solution close to the CNP surface responds to the changes in the potential. When the frequency is low, both the ion transport and the electrochemical reaction in the entire volume can follow the sinusoidal perturbation, producing a charge saturation that is transduced into an abrupt increase in Z_{IM} in the Nyquist plot.[15] It is worth noting that, in EIS measurements of nanofluidic devices containing redox probes, the semicircle typically associated with charge transfer resistance (kinetic control) is not clearly distinguishable. This is because the magnitude of the charge transfer resistance is significantly lower than that of the ion transport resistance, and both processes occur within similar characteristic frequency ranges, making it difficult to resolve them separately in the impedance spectra. It is worth mentioning that, at frequencies higher than the pure capacitive trend of the charge saturation regime, some systems can also exhibit a 45° linear trend due to a diffusion-controlled behavior.[8,9] However, depending on the specific case, such a 45° linear trend can be small enough and, consequently, difficult to be evidenced in the EIS output.[8,10] For example, certain already reported results under a thin-layer regime observed an increase in the characteristic frequency of the charge saturation region as the solution layer decreases, producing a shortening in the 45° linear trend.[11] Considering these antecedents, we hypothesize that the diffusion region is not evidenced in our experiments because the cross-section of the nanopipette is in the order of hundreds of nanometers, and, consequently, such a diffusion control must overlap with the kinetic control region.[12]

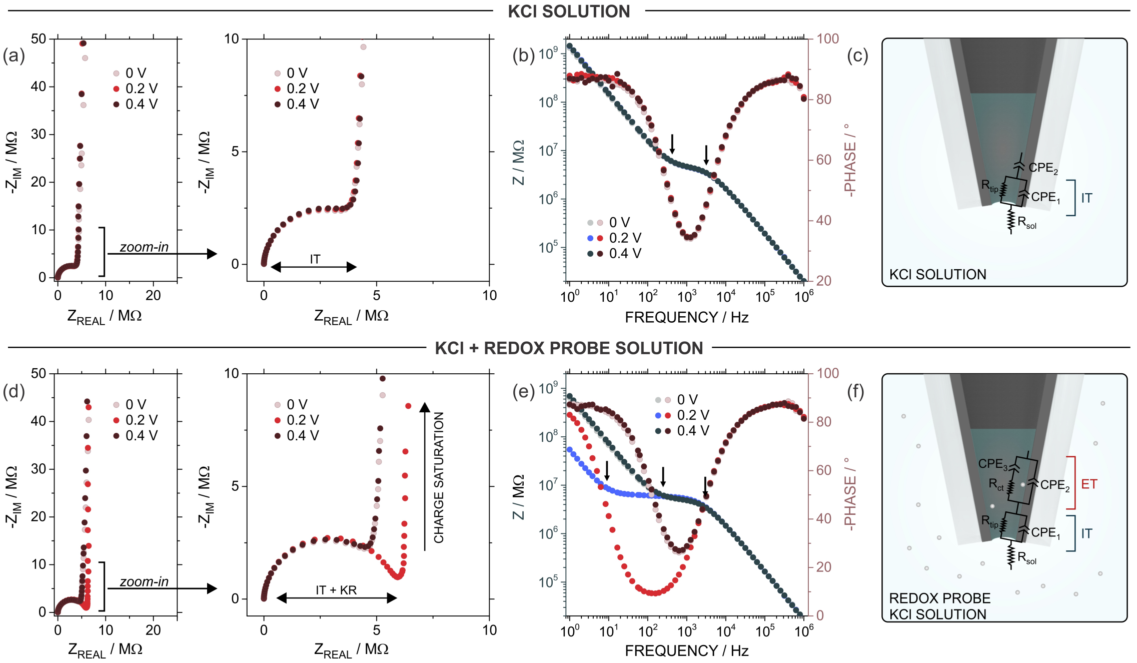

Figure S6. (a) Nyquist and (b) Bode plots at different E_{DC} for measurements conducted in a 0.3 M KCl solution. (c) Scheme of the proposed equivalent circuit for the system in the absence of any redox reaction. (d) Nyquist and (e) Bode plots at different E_{DC} for measurements performed in a solution containing 0.3 M KCl + 7.7 x 10^{-4} M $Fe(II)(CN)_6^{4-}$. (f) Scheme of the proposed equivalent circuit for the system in the presence of redox reactions. ET and IT refer to electron transfer and ion transport, respectively. KR refers to the kinetic region of the electron transfer reaction. The arrows in the Bode Plot indicate the breaking points.

EIS of different CNP geometries

Variations in tip dimensions and pipette geometry have a significant impact on the semicircle diameter observed in EIS measurements. For example, CNPs with tip diameters of approximately 100 nm and 1000 nm exhibited ion resistances of around 7 MΩ and 1 MΩ, respectively. For this kind of device, the ion resistance (R_{CNP}) can be related to the tip diameter (d_{tip}) and the cone angle (θ) according to the following expression:[13]

$$R_{CNP} = \frac{2}{\kappa d_{tip}}(\frac{1}{\pi tan\theta} + \frac{1}{4})$$

These experimentally observed differences in ion resistance are consistent with the expected order of magnitude predicted by the equation. However, for a more precise analysis, accurate knowledge of the cone angle is also required.

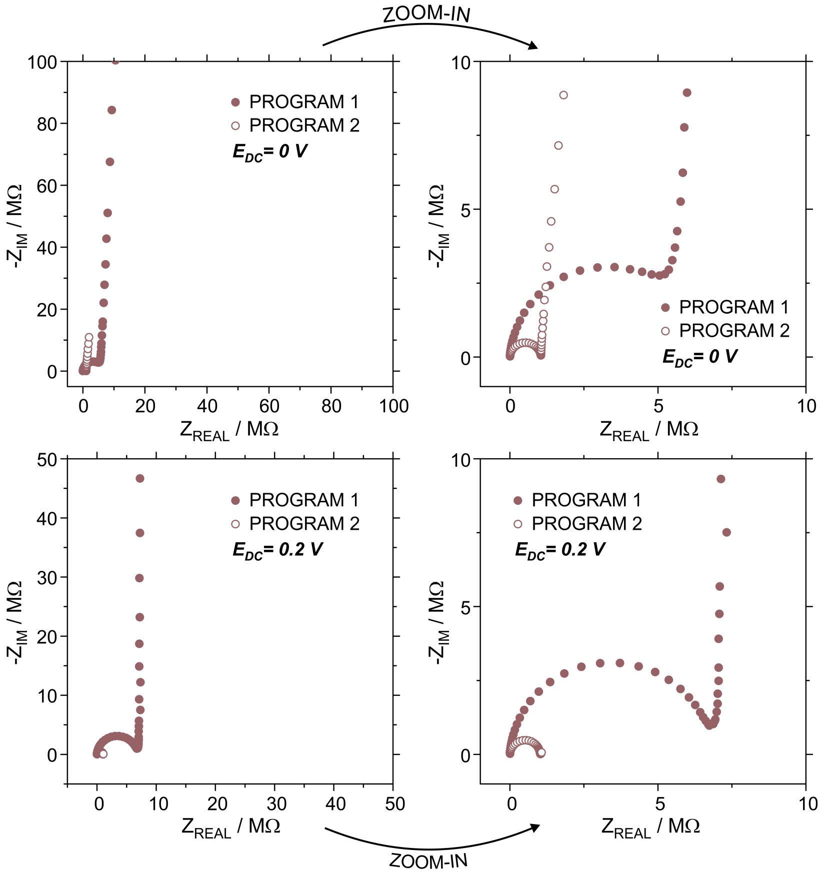

Figure S7. Nyquist plots at (a) E_{DC}= 0 V and (b) E_{DC}= 0.2 V for three carbon pipettes with different tip sizes.

Electrochemical response at different supporting electrolyte concentrations

EIS results have demonstrated the crucial role of ion transport in the electrochemical performance of the developed CNP. To further probe this influence, additional electrochemical experiments were carried out in media of varying ionic resistances by systematically altering both the concentration and the nature of the supporting electrolyte. The CV and EIS measurements were conducted at a fixed ferrocyanide concentration (7.7 x 10^{-4} M), while the KCl concentration was varied from 0.3 M to 1.5 M. The results are depicted in **Figure S8** and **S9**. The KCl concentration range was selected to minimize any surface charge effect, which tends to arise at lower concentrations (< 0.1 M).[14,15] This was supported by the observed CVs (**Figure S8a**), which displayed minimal variations in the current readout at varying KCl concentrations. Notably, as the experimental setup consisted of an Ag/AgCl wire as the reference electrode, the voltammetric peaks shifted towards higher voltage values as the Cl^{-} concentration was increased.

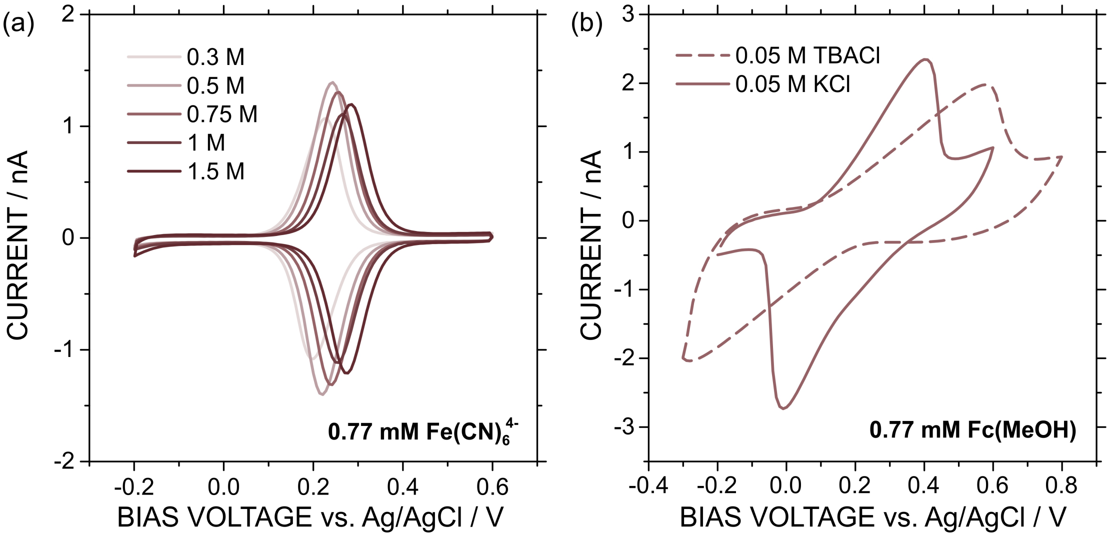

Figure S8. CVs at different supporting electrolyte conditions. (a) CVs at varying KCl concentrations, with the $Fe(CN)_6^{4-}$ redox probe concentration fixed at 0.77 mM. (a) CVs obtained using either 0.05 M tertbutylammonium chloride (TBACl) or 0.05 M KCl as the supporting electrolyte, with the ferrocene methanol (Fc(MeOH)) redox probe concentration fixed at 0.77 mM.

EISs were recorded at two different E_{DC}= 0 V and $E^{0'}$(in all the cases, the formal potential was extracted from the CV curves at the different supporting electrolyte concentrations, see **Materials & Methods** for further details). The analysis of the corresponding Nyquist plots evidenced a clear incidence of the electrolyte concentration in the CNP ion resistance (**Figures S9a-i** and S**9a-ii**). Specifically, as the concentration increased, -Z_{IM} and Z_{REAL} decreased, reducing the size of the semicircle. This indicated a diminution in the tip ion resistance.[16] The Bode plots also showed a diminution in $|Z|$ as the concentration increased, which is correlated to the mentioned decrease in the resistance (**Figure S9a-iii** and **S9a-iv**). For instance, an increment of KCl concentration from 0.3 M to 1 M decreased Z_{REAL} (at 1500 Hz) from 4.5 MΩ to 1.6 MΩ. On the other hand, the phase angles did not display significant changes at the different concentrations.

Beyond the concentration, the ion resistance in the tip is drastically affected by the nature of the supporting electrolyte and, consequently, the mobility of the ions involved in the transport. CVs and EISs were obtained in either 0.05 M KCl or 0.05 M tetrabutylammonium chloride (TBACl) as the supporting electrolyte to evaluate this effect (**Figure S9b**). The selected concentration is limited by the solubility of TBACl in water. Notably, the experiments were conducted using the redox probe ferrocene methanol (FcMeOH, 7.7x10^{-4} M), as the surface charge effects are less pronounced for this one compared to those expected for highly charged redox couples as $Fe^{[II]/[III]}(CN)_6^{4-/3-}$.[14]

The Nyquist plots revealed a similar effect as that previously explained at different KCl concentrations (**Figure S9b-i** and **S9b-ii**). At E_{DC}= 0 V and $E^{0'}$, the replacement of K^+ by TBA^+ ions generated a significant increment of 2.5 times the semicircle diameter in the Nyquist plot. This fact is due to the increment in the ion resistance, which can be ascribed to the low diffusion coefficient (*D)* of the TBA^+ (and consequently, ion mobility) compared to the K^+ ions, i.e., $D(TBA^+)=0.52\times10^{-5}$ cm^2 s^{-1} vs. $D(K^+)1.96\times10^{-5}$ cm^2 s^{-1})[17].[16] Conversely to the behavior exhibited at different KCl concentrations, the Bode plots demonstrated a significant shift in the phase angle plot towards lower frequencies and, a wide frequency-independent */Z/* region for the experiments performed in TBA^+. This led to a variation in the characteristic times, as evidenced by the decrease of the high-frequency breaking point from 260 Hz (0.0038 s) in KCl to 89 Hz (0.011 s) in TBA^+ (**Figure S9b-iii** and **S9b-iv**). The results indicated that salts composed of ions with low mobilities increase the ion resistance and also delay the processes involved during the electrochemical reaction. This fact directly affects the response obtained in the CV, generating an increase in the peak separation as well as wider peaks (**Figure S8b**).

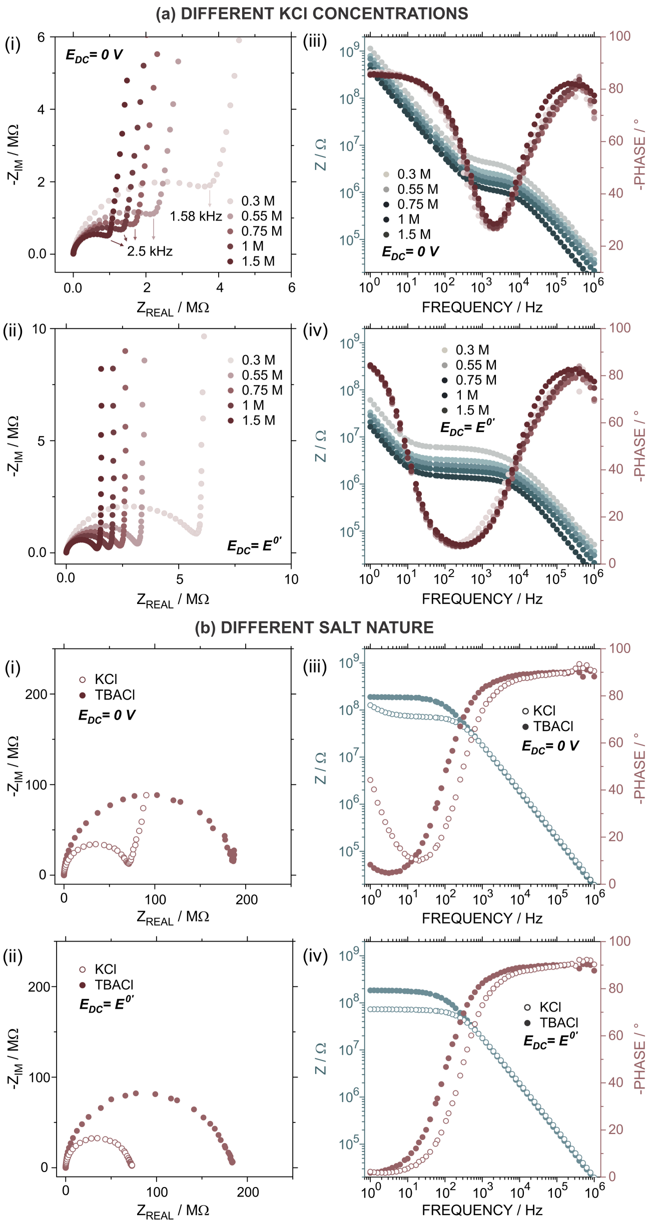

Figure S*9*. (a) EIS measurements at various solutions comprising 7.7 x 10^{-4} M $Fe^{[II]}(CN)_6{}^{4-}$ and different KCl concentrations. (b) EIS measurements using 0.05 M of KCl or TBACl as the supporting electrolyte and 7.7 x 10-4 M of ferrocene methanol as the redox probe. Figures (i) and (ii) show the Nyquist and Bode plots at E_{DC}=0. Figures (iii) and (iv) show the Nyquist and Bode plots at $E_{DC}=E^{0'}$.

EIS response in the presence and absence of redox probe

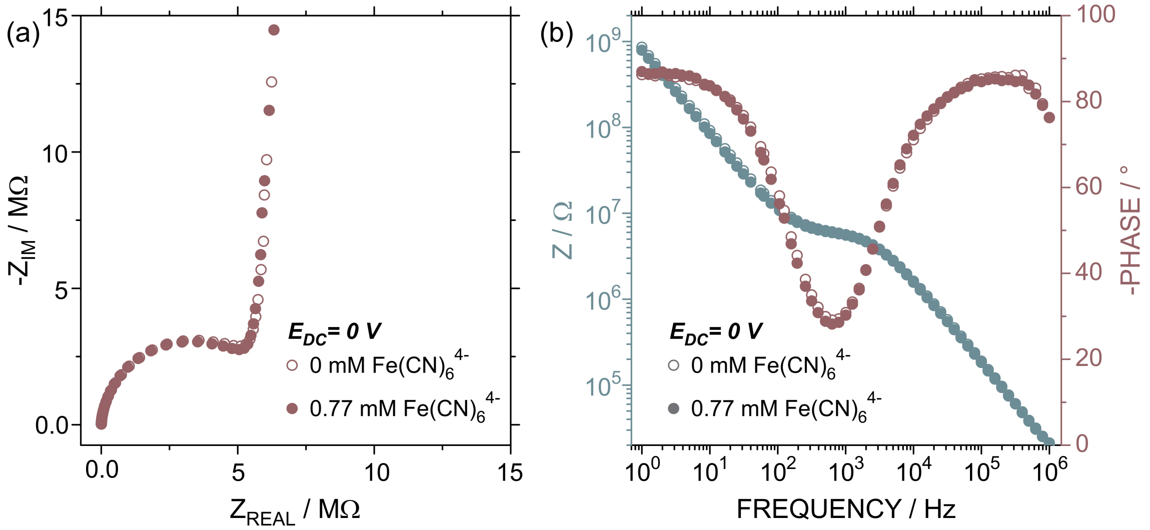

Figure S10. (a) Nyquist and (b) Bode plots obtained at E_{DC}= 0 V in the presence of a solution of 0.3 M KCl without any redox probe (void circles) and with 0.77 mM of $K_4Fe(CN)_6$ (full circles).

Equivalent circuit analysis

The medium-high frequency signal was modeled by a simplified Randless equivalent circuit (EC) with the solution resistance (R_{sol}) in series with an (RC) component, where R_{tip} and C_1 represent the CNP resistance and capacitance $R_{sol}(R_{tip}C_1)$. These components predict the semicircle in the Nyquist plot.

The abrupt increase of Z_{IM} at low frequencies is characteristic of a capacitive behavior (C_2) related to the electrical double-layer charging. This charging current arises from the external voltage applied to the carbon walls. For this reason, it does not appear in ion transport experiments. Such a trend was modeled by including a capacitor in series to the $R_{sol}(R_{tip}C_1)$ component. Considering the deviations from the ideal capacitive behavior, which could arise due to the carbon layer porosity and inhomogeneity, as well as the asymmetric geometry of the pipette, constant phase elements (*CPE*) were employed in the fitting instead of pure capacitors.[18–20] However, it is worth noting that while the "n" parameter of the nanofluidic CPE (CPE_1) was approximately 0.9, the CPE associated with the electrical double layer and the charge saturation region exhibited an n value >0.95, indicating an almost ideal capacitive behavior (**Table S2** and **S3**).

Figure S11 presents the fittings of the EIS responses at E_{DC}=0 and 0.4 V to the ECs $R_{sol}(R_{tip}CPE_1)CPE_2$ and $R_{sol}([R_{tip}CPE_2]CPE_1)$ for a CNP sample measured in a solution containing 0.3 M KCl + 7.7 x 10^{-4} M $Fe^{(II)}(CN)_6^{4-}$. Due to the higher instrumental error in the data obtained at very low (<10 Hz) and high frequencies (>10^5 Hz) (Kramers-Kronig analysis, **Figure S5**), the fittings were conducted in the frequency window of 10 Hz – 10^5 Hz. This selection enabled a 10-fold diminution of χ^2 without significant variations in the magnitudes of the parameters involved in the EC. Nevertheless, both approaches acceptably reproduced the experimental results with similar values for the circuit components, with fittings displaying 0.01> χ^2.

At $E_{DC}=E^{0'}$, an additional contribution from the electron transfer of the redox probe to the carbon layer is observed, alongside ion transport through the CNP. To account for this situation, the experimental data were fitted using the same models previously described, with the inclusion of an [RCPE] component representing the charge transfer resistance (R_{ct}) and the charge saturation capacitance (CPE_3), respectively (**Figure S6f**). Like the cases at 0 and 0.4 V, the quality of the fit in terms of Z_{IM} and Z_{REAL} errors and χ^2 (<0.01) was rather adequate (**Figure S12**). Notably, further efforts and analyses (e.g., other EC fittings, DRT)[21] may provide more fundamental information on the system, which could be addressed in future research directions.

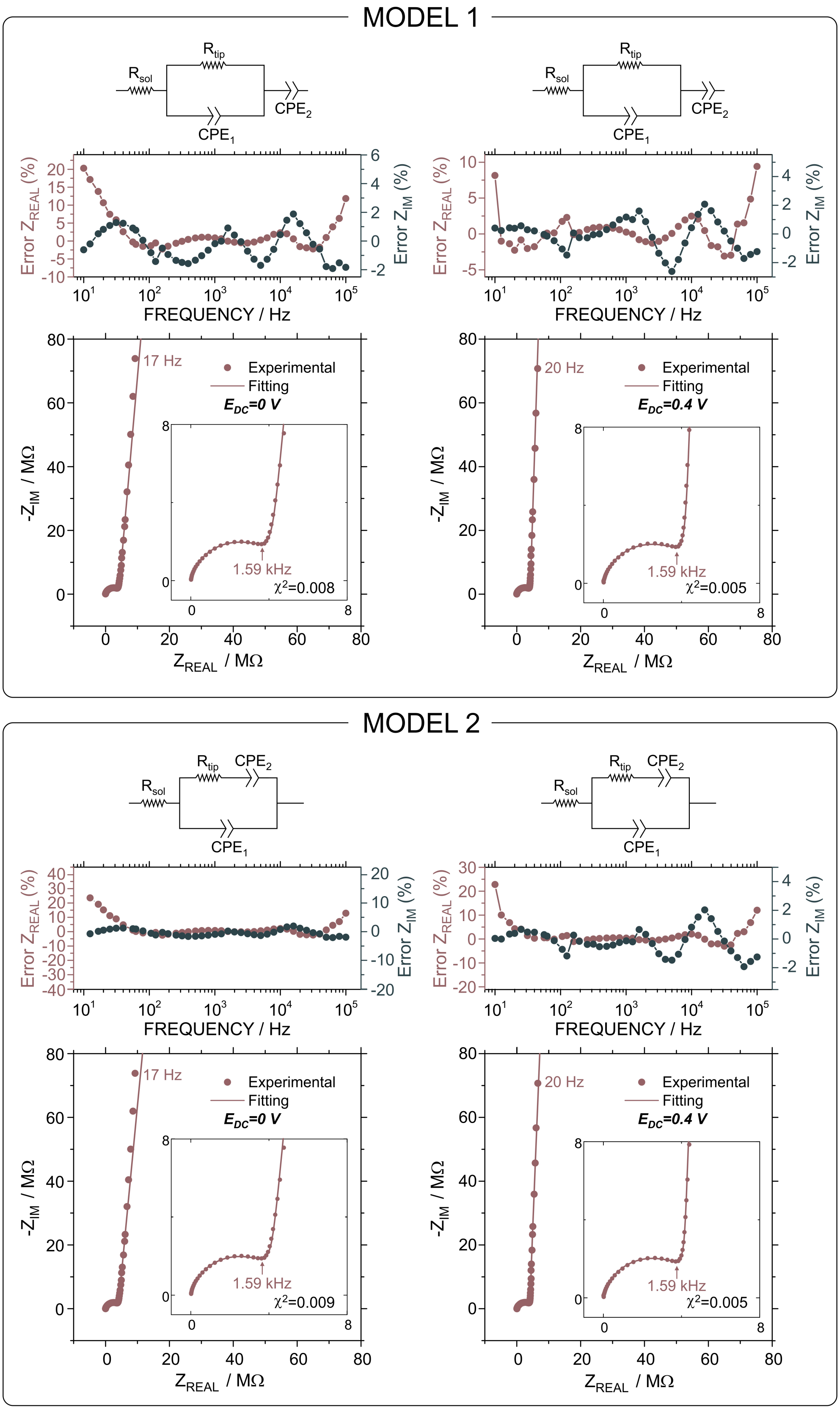

Figure S11. Experimental Nyquist plots at E_{DC}=0 V (left panels) and 0.4 V (right panels) and their fitting to two different equivalent circuit models. Model 1 (top panel): $R_{sol}(R_{tip}//CPE_1)CPE_2$; Model 2 (bottom panel): $R_{sol}(R_{tip}CPE_2//CPE_1)$. The figure shows the percentual error in both components of Z for the different frequencies.

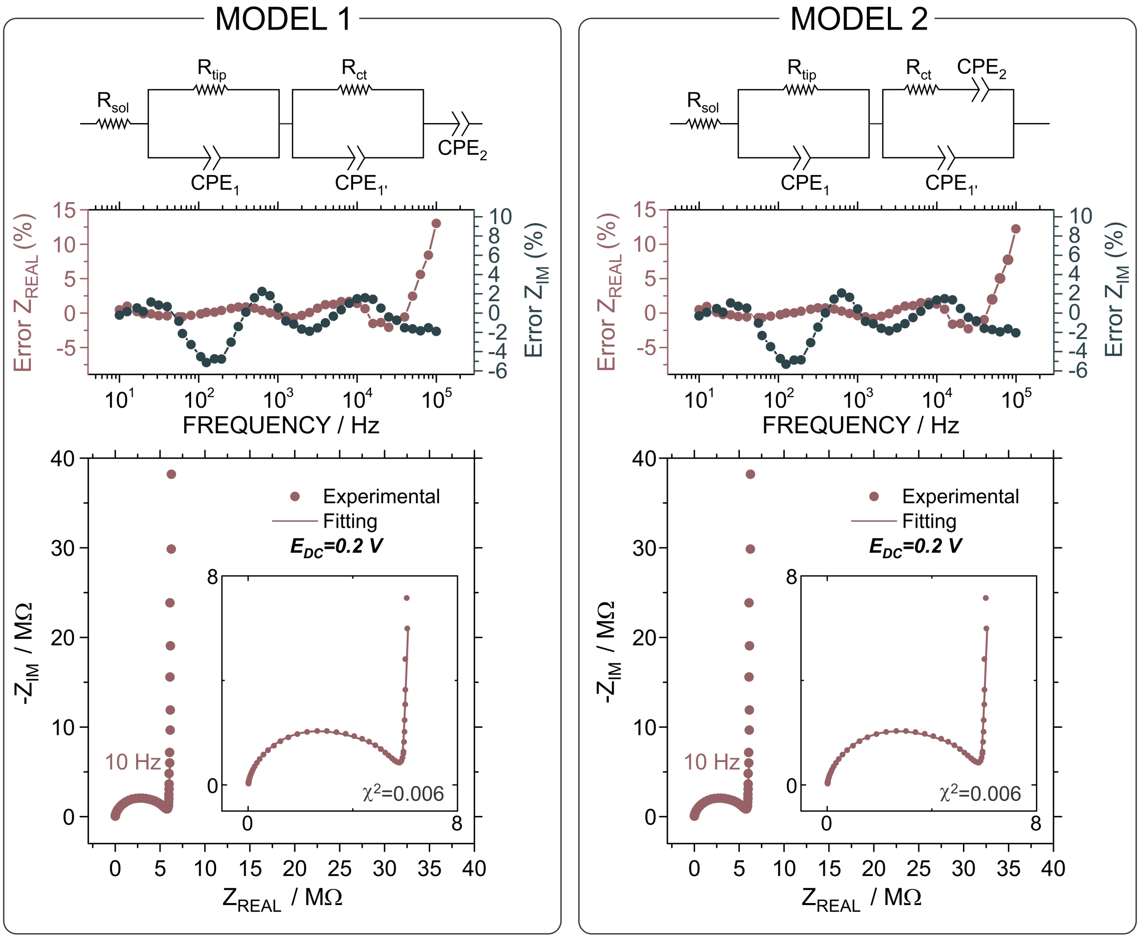

Figure S12. Experimental Nyquist plots at E_{DC}=0.2 V and their fittings to two equivalent circuit models denoted as MODEL 1 and MODEL 2. The figure shows the percentual error in both components of Z for the different frequencies.

Cyclic voltammetries and EIS at different redox probe concentrations

Experimental results in **Figure S13** only show slight changes in the peak potentials with the scan rate and ferrocyanide concentration, which can be attributed to the less influence of ion resistance on the CVs when the experiments are performed in CNP with a low filling degree (see below). In contrast, for CNPs with higher solution depths, it is expected an accentuation in ΔE_p when the redox probe concentration is increased, due to limitations given by the ion transport. To further clarify this point, the effect of the volume at different redox probe concentrations was evaluated by numerical simulations (**Figure S14**). While a 50 μm solution depth resulted in ΔE_p variations of less than 10 mV (at 50 mV s^{-1}), increasing the depth to 100 μm raised the value for ΔE_p up to 80 mV. This effect becomes even more pronounced at higher scan rates.

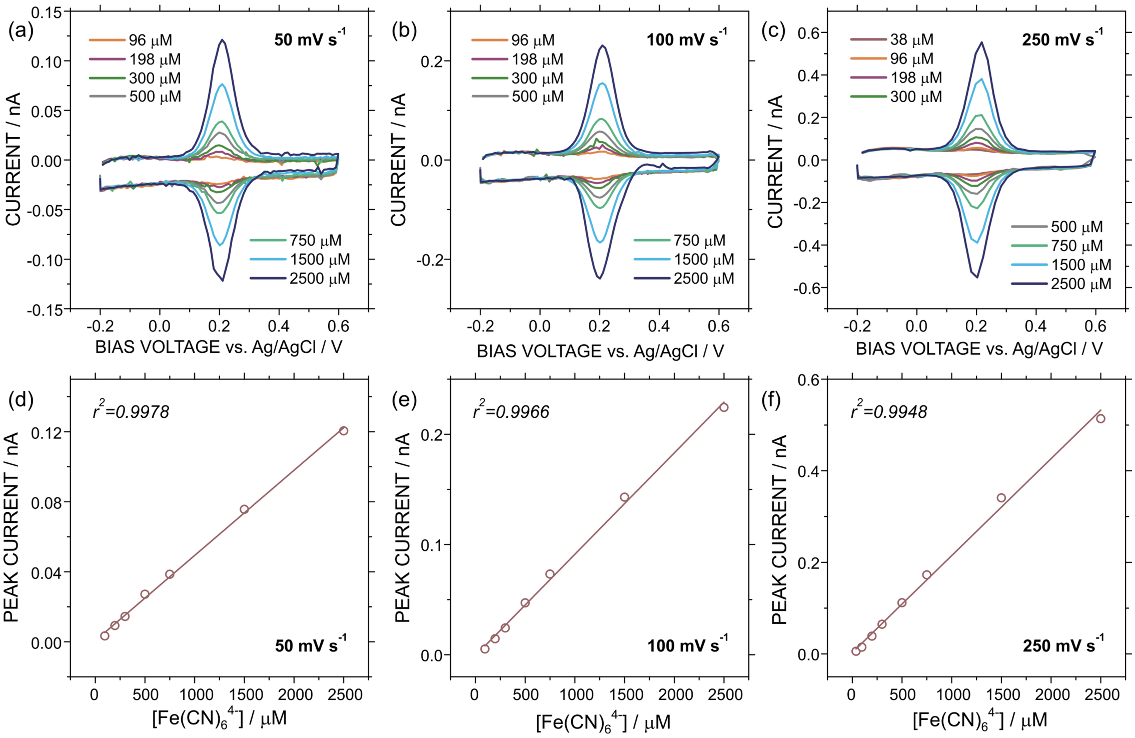

Figure S13. Experimental cyclic voltammetries at different ferrocyanide concentrations ($[Fe(CN)_6^{-4}]$) for v= (a) 50 mV s^{-1}, (b) 100 mV s^{-1} and (c) 250 mV s^{-1}. Anodic peak current in terms of $[Fe(CN)_6^{-4}]$ for v= (d) 50 mV s^{-1}, (e) 100 mV s^{-1} and (f) 250 mV s^{-1}. All the measurements were carried out employing 0.3 M KCl as the supporting electrolyte.

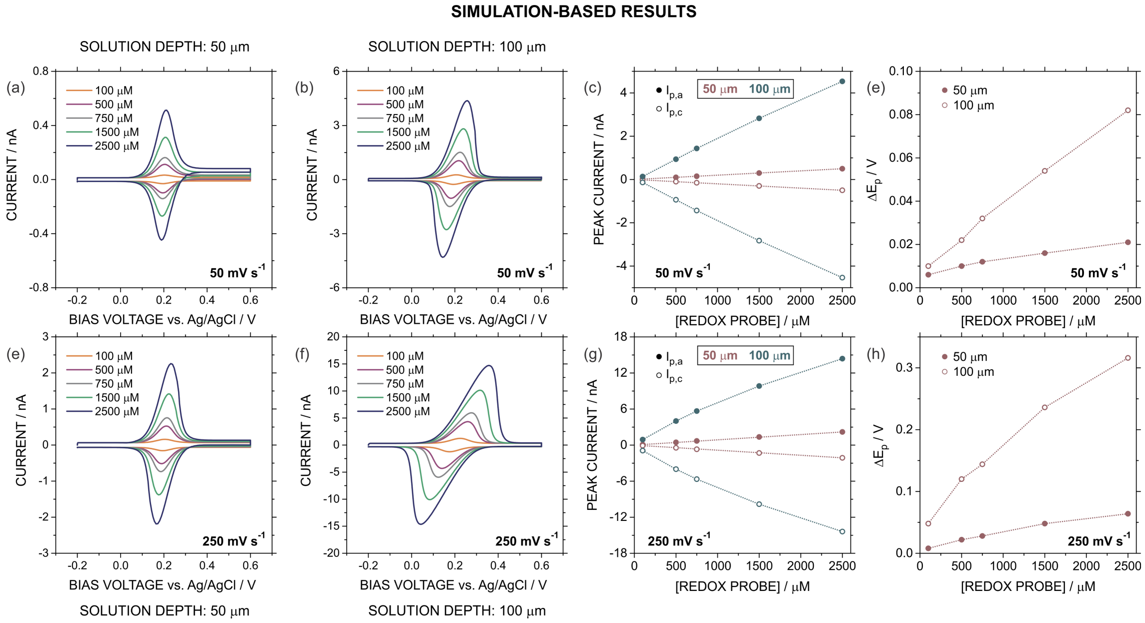

Figure S14. Simulated CVs at different redox probe concentrations (v= 50 mV s−1) for a CNP with solution depth of (a) 50 μm and (b) 100 μm. (c) Anodic ($I_{p,c}$) and cathodic peak currents ($I_{p,c}$) in terms of [Fe(CN)$_6$$^{-4}$] for the voltammograms obtained at both solution depth and v= 50 mV s-1. (d) Peak potential separation (ΔE_p) in terms of [Fe(CN)$_6$$^{-4}$] for the voltammograms obtained at both solution depth and v= 50 mV s−1. CVs at different redox probe concentrations (v= 250 mV s−1) for a CNP with solution depth of (e) 50 μm and (f) 100 μm. (g) Anodic ($I_{p,c}$) and cathodic peak currents ($I_{p,c}$) in terms of [Fe(CN)$_6$$^{-4}$] for the voltammograms obtained at both solution depth and v= 250 mV s−1. All the simulations were carried out by considering a CNP with a radius of 50 nm, tan(θ)=0.2, capacitance of 20 μF/cm^2, and 0.3 M KCl as the supporting electrolyte.

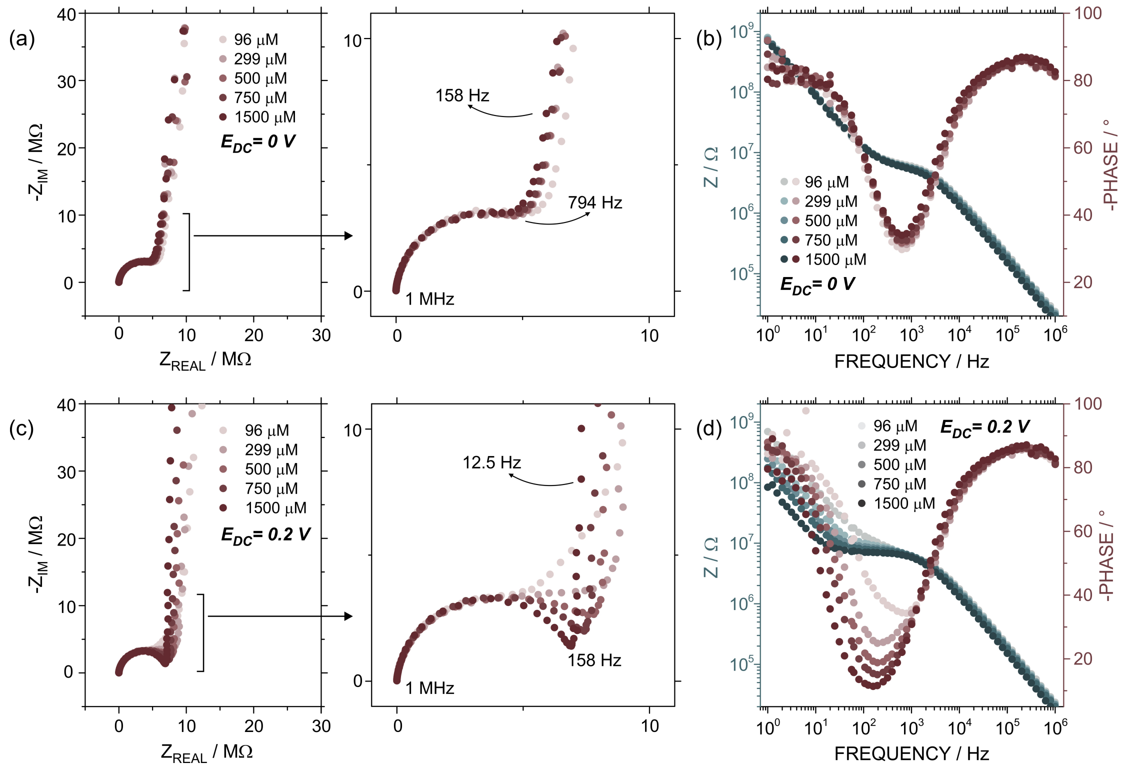

Figure S15. (a)(c) Nyquist and (b)(d) Bode plots obtained at E_{DC}= 0 V and 0.2 V, respectively, for different ferrocyanide concentrations.

Electrochemical response at different inner volumes.

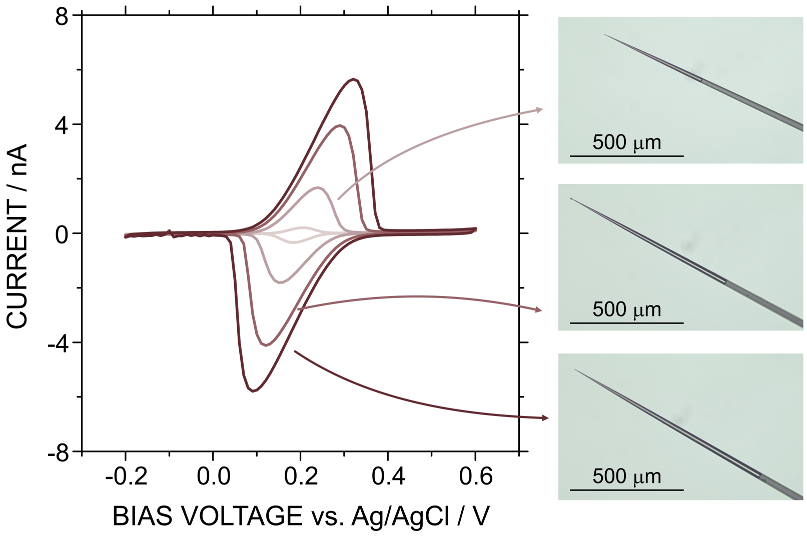

Figure S16. CVs for a CNP with different solution depths. Optical microscopy images denote the different solution volumes inside CNPs after each CV measurement. The CV plot with the lowest current magnitudes represents the initial measurement. All the measurements were carried out in a solution 0.3 M KCl + 7.7 x 10^{-4} M $Fe(CN)_6^{4-}$.

Figure S17 presents the performance of a CNP in 0.3 M KCl + 0.77 mM $K_4Fe^{[II]}(CN)_6$ at three solution depths evaluated in turn at increasing *v*. For very low volumes (e.g., 3.7 pL), the peak current evidenced a linear growth with *v* that accounts for the maintenance of the thin-layer regime confinement (**Figure S17a**). If the volume inside the CNPs is increased (e.g., 0.48 nL), the current is attenuated at high scan rates, losing the linear relationship. In contrast, I_c not only increases with the volume but also maintains a linear relationship with *v* (**Figure S17b**). This fact leads to a decrease in the I_p / I_c ratio as *v* increases, if the volume inside the CNP is high enough, demonstrating another clear difference with the behavior obtained at low solution depth. For instance, the I_p / I_c ratio at 0.48 nL decreased from 90 at 10 mV s^{-1} to 45 at 250 mV s^{-1}. A similar effect occurred with the peak potentials, where the increment in the peak separation obtained at high volumes is strongly accentuated when *v* is increased (**Figure S17c**). For example, the peak separation at 50 mV s^{-1} increases from 80 mV to 300 mV with an increment in the volume from 0.48 nL to 1.33 nL. However, at 250 mV s^{-1}, these values are 200 mV and 730 mV, respectively. While all these results significantly disobey the basic fingerprints of the thin-layer behavior, *Q* is still maintained with *v* for the three evaluated filling degrees (<10% of variation, **Figure S17d**). This fact demonstrates the total consumption of the analyte during the CV timescale, which supports the use of the voltammetric charge for coulometric determination in a wide range of volumes and scan rates.

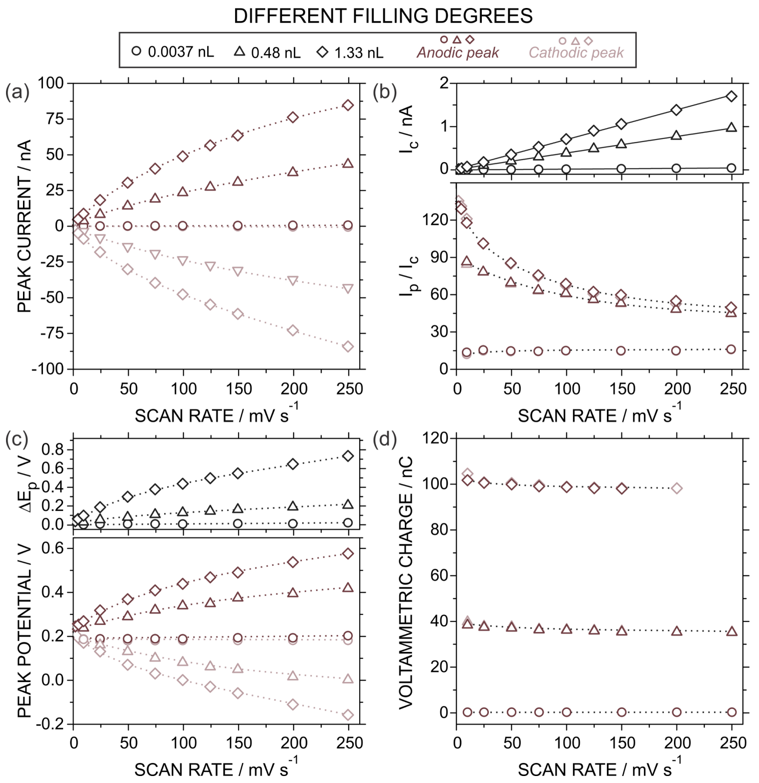

Figure S17. (a) Plot of the peak current against the scan rate for different solution volumes. (b) Plots of the capacitive current (top) and I_p/I_c ratio (bottom) against the scan rate for different solution volumes. (c) Plots of the peak potential separation (top) and peak potentials (bottom) at increasing scan rate for different solution volumes. (d) Plot of the voltammetric charge for the anodic (red) and cathodic (blue) peaks at increasing scan rate for a CNP with different solution volumes. All the measurements were conducted in 0.3 M KCl + 7.7x10^{-4} M $Fe^{[II]}(CN)_6^{4-}$.

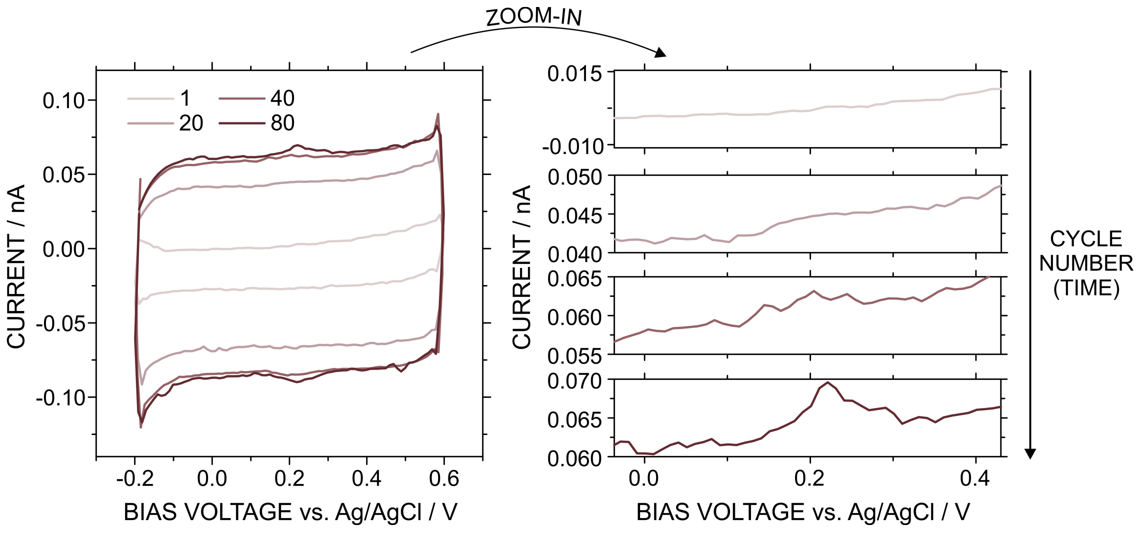

Figure S18. CVs for a CNP in a solution 0.3 M KCl + 7.7 x 10^{-7} M $Fe(CN)_6^{4-}$ at scan rate 50 mV s^{-1}. The legend indicates the cycle number.

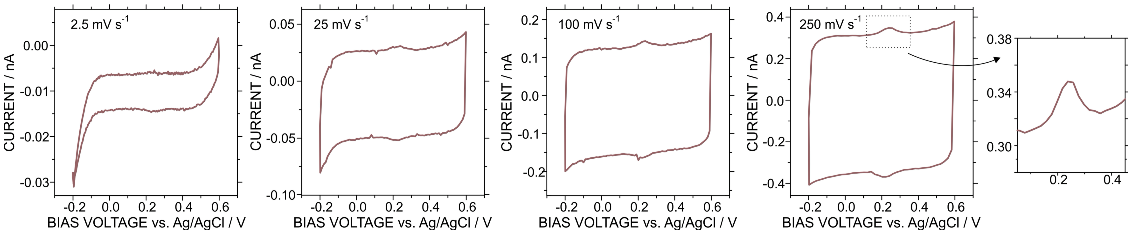

Figure S19. CVs at different scan rates for a CNP in a solution 0.3 M KCl + 7.7 x 10^{-7} M $Fe(CN)_6^{4-}$.

All these results, together with the analysis in Figures 4 and 5, suggest that there are several variables to optimize for achieving the best signal-to-noise ratio. In this case, we will focus on the volume and scan rate.

For a given concentration of redox analyte, the maximization of the volume sensitively increments the signal-to-noise ratio (both in terms of current and charge). This is at the expense of an increment in the width and separation of the peaks, which, in the case of samples containing more than one redox moiety, could affect the resolution. For these cases, high supporting electrolyte concentrations could decrease the peak separation.

Increasing the scan rate also enables the increment of the current. In those cases where the signal is appreciable, its increment is not transduced into an increment of I_p/I_c, as shown in **Figure 3b**. However, for very diluted samples where I_p is very low (unity of pA) and I_c fairly exceeds its magnitude, the increment in the scan rate could facilitate the determination of I_p (and Q) and, thus, enhance the detection, as shown in **Figure S19**.

Finally, it is worth noting that in all cases, the detection limit of the device could be further improved by tailoring the surface properties. Previous studies have shown that analyte adsorption and electrostatic interactions with surface functional groups can enrich—or, in some cases, deplete—the local concentration of molecules or ions within the nanofluidic device.[14,22]

The analyzed trends in **Figure 4** show that the CNP electrochemistry is sensitively affected by the ion transport through the tip of its nanofluidic structure. Effectively, the application of a bias voltage triggers the migration of ions (cations towards the cathode and anions towards the anode). In addition, the electrochemical reaction of the redox probe generates a local charge imbalance that requires a flux of additional ions from the supporting electrolyte to maintain electroneutrality. When the number of moles reacting into the nanopipette is high, the time involved in the overall process starts to be limited by the ion fluxes through the tip, which is in turn translated to a distortion and shift of the peak potentials. This effect is illustrated in **Figure S20** through numerical simulations, which were performed under two different theoretical frameworks: (i) considering a potential gradient where the charge is balanced due to the supporting electrolyte transport (i.e., including tip and solution resistance), and (ii) under ideal conditions (i.e., without any uncompensated resistance from the tip and solution). In this latter case (considering no ion resistance), the redox peaks in the CV exhibited the typical symmetric Gaussian profile expected for thin-layer electrochemical reactions. In contrast, when the tip and solution resistances are considered, the CV showed certain peak deformation, particularly at higher solution depths, which negatively impacts the I_p magnitude and increases the E_p. Beyond the peak distortion, as mentioned above, the total analyte consumption was achieved in all cases. As a result, the peak charge remains unchanged in the CVs, regardless of the model used.

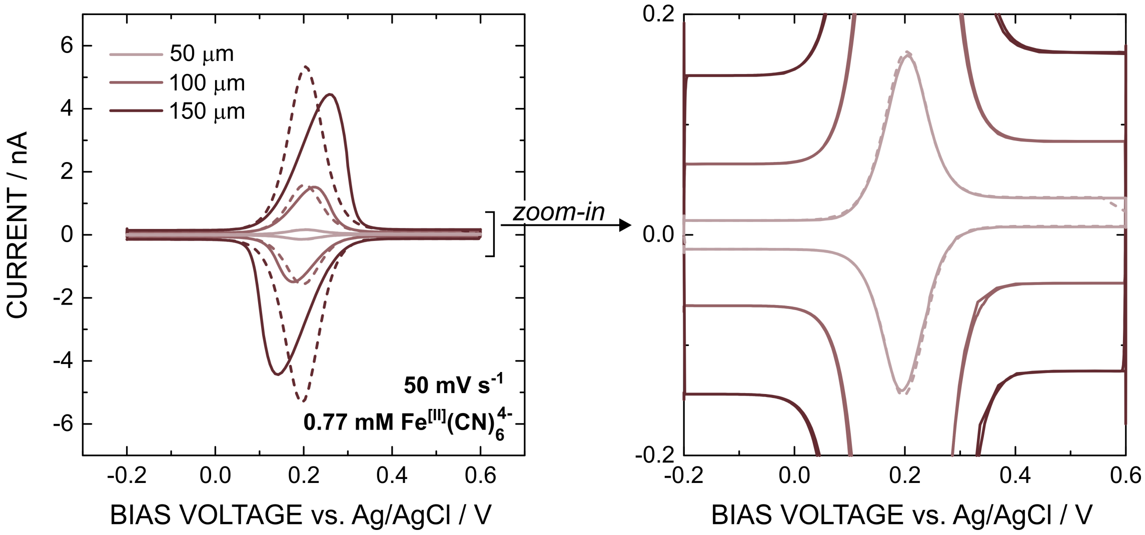

Figure S20. Theoretical CVs at 50 mV s^{-1} estimated at different solution depths. Dashed lines represent model predictions excluding potential gradients (ideal case without considering tip resistance contributions), while solid lines correspond to model results accounting for ion transport effects. All the simulations were carried out by considering a CNP with a radius of 50 nm, tan(θ)=0.2, a capacitance of 20 $\mu F/cm^2$, and a solution composed of 0.3 M KCl + 7.7 x 10^{-4} M $Fe(CN)_6^{4-}$.

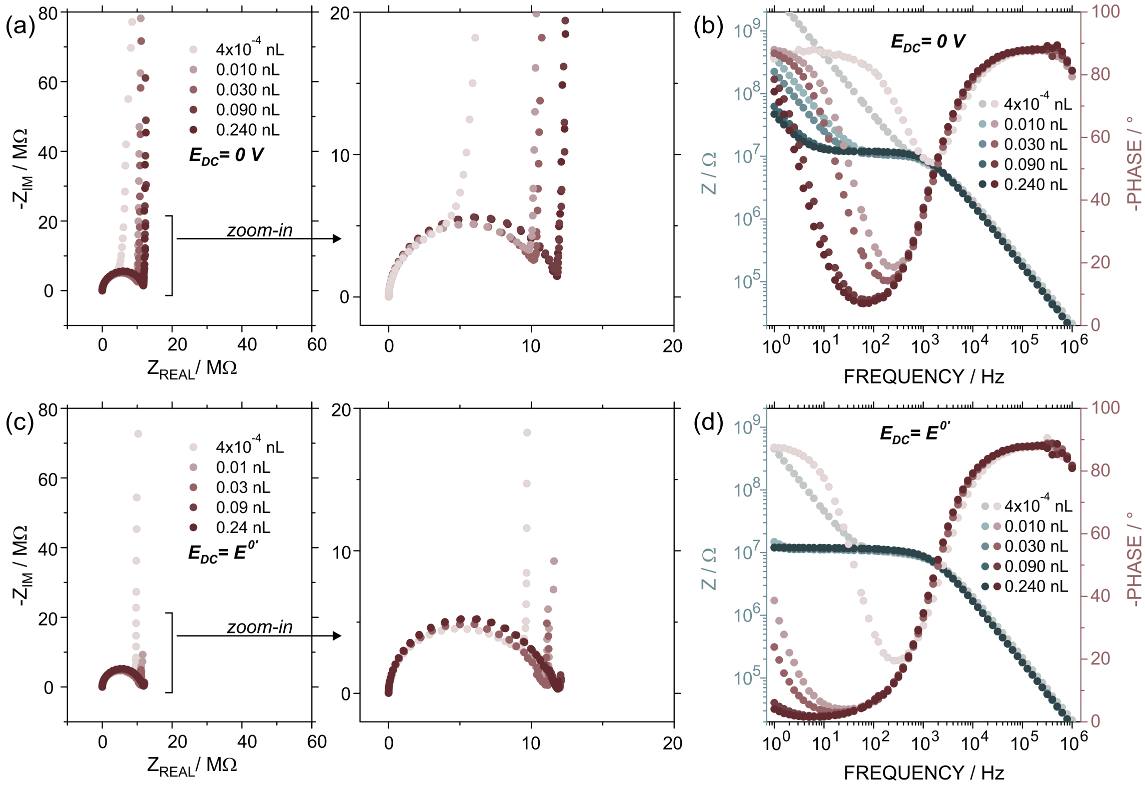

Figure S21. (a) Nyquist and (b) Bode plots for a CNP with different solution volumes at E_{DC}= 0 V. (c) Nyquist and Bode plots for a CNP with different solution volumes at E_{DC}= $E^{0'}$= 0.2 V. All the measurements were performed in a mixture of 0.3 M KCl + 7.7 x 10-4 M $Fe[II](CN)_6^{4-}$.

4. Additional tables

Table S2. Results of the fittings to two different equivalent circuits of the EIS spectra obtained at E_{DC}=0 V.

PARAMETER		MODEL 1		MODEL 2	
		0 V	0.4 V	0 V	0.4 V
R_{sol} / kΩ		5.91	5.91	5.90	5.90
R_{tip} / MΩ		4.09	4.20	4.66	4.77
CPE_1	Y_0 / $s^N.\Omega^{-1}$	2.01 x 10^{-11}	1.85 x 10^{-11}	1.93 x 10^{-11}	1.84 x 10^{-11}
	N	0.88	0.88	0.88	0.88
CPE_2	Y_0 / $s^N.\Omega^{-1}$	1.65 x 10^{-10}	1.23 x 10^{-10}	1.52 x 10^{-10}	1.10 x 10^{-10}
	N	0.95	0.98	0.94	0.98
χ		0.008	0.005	0.009	0.005

R_{sol} was fitted for MODEL 1 – 0 V, and then, it was fixed for the rest of the tests. However, variations in this parameter between 0.5 and 6 kΩ do not generate substantial variations in the rest of the magnitudes (< 10%).

The fitting was performed from 10 Hz to 100 kHz.

Table S3. Results of the fittings to two different equivalent circuits of the EIS spectra obtained at E_{DC}=0.2 V.

PARAMETER		MODEL 1	MODEL 2
		0.2 V	0.2 V
R_{sol} / kΩ		5.91	5.90
R_{tip} / MΩ		4.14	4.04
CPE_1	Y_0 / $s^N.\Omega^{-1}$	2.15 x 10^{-11}	2.13 x 10^{-11}
	N	0.875	0.88
R_{ct} / MΩ		1.75	1.99
$CPE_{1'}$	Y_0 / $s^N.\Omega^{-1}$	1.60 x 10^{-10}	1.67 x 10^{-10}
	N	0.95	0.90
CPE_2	Y_0 / $s^N.\Omega^{-1}$	2.948 x 10^{-9}	2.818 x 10^{-9}
	N	0.98	0.98
χ		0.006	0.006

5. References

(1) Singhal, R.; Bhattacharyya, S.; Orynbayeva, Z.; Vitol, E.; Friedman, G.; Gogotsi, Y. Small Diameter Carbon Nanopipettes. *Nanotechnology* **2010**, *21* (1), 015304. https://doi.org/10.1088/0957-4484/21/1/015304.

(2) Aref, M.; Ranjbari, E.; García-Guzmán, J. J.; Hu, K.; Lork, A.; Crespo, G. A.; Ewing, A. G.; Cuartero, M. Potentiometric PH Nanosensor for Intracellular Measurements: Real-Time and Continuous Assessment of Local Gradients. *Anal. Chem.* **2021**, *93* (47), 15744–15751. https://doi.org/10.1021/acs.analchem.1c03874.

(3) Liu, R.; Ma, Y.; Shen, X.; Wang, D. Quantification of the Charge Transport Processes inside Carbon Nanopipettes. *Chem. Sci.* **2021**, *12* (44), 14752–14757. https://doi.org/10.1039/D1SC04282C.

(4) McDermott, M. T.; McDermott, C. A.; McCreery, R. L. Scanning Tunneling Microscopy of Carbon Surfaces: Relationships between Electrode Kinetics, Capacitance, and Morphology for Glassy Carbon Electrodes. *Anal. Chem.* **1993**, *65* (7), 937–944. https://doi.org/10.1021/ac00055a017.

(5) Xu, J.; Granger, M. C.; Chen, Q.; Strojek, J. W.; Lister, T. E.; Swain, G. M. Peer Reviewed: Boron-Doped Diamond Thin-Film Electrodes. *Anal. Chem.* **1997**, *69* (19), 591A-597A. https://doi.org/10.1021/ac971791z.

(6) Yu, Y.; Noël, J.-M.; Mirkin, M. V.; Gao, Y.; Mashtalir, O.; Friedman, G.; Gogotsi, Y. Carbon Pipette-Based Electrochemical Nanosampler. *Anal. Chem.* **2014**, *86* (7), 3365–3372. https://doi.org/10.1021/ac403547b.

(7) Wang, S.; Zhang, J.; Gharbi, O.; Vivier, V.; Gao, M.; Orazem, M. E. Electrochemical Impedance Spectroscopy. *Nat. Rev. Methods Prim.* **2021**, *1* (1), 41. https://doi.org/10.1038/s43586-021-00039-w.

(8) Vivier, V.; Orazem, M. E. Impedance Analysis of Electrochemical Systems. *Chem. Rev.* **2022**, *122* (12), 11131–11168. https://doi.org/10.1021/acs.chemrev.1c00876.

(9) Bard, A. J.; Faulkner, L. R. *Electrochemical Methods. Fundamentals and Applications*, 2nd ed.; Harris, D., Swain, E., Eds.; Wiley: USA, 2001.

(10) Mei, B.-A.; Munteshari, O.; Lau, J.; Dunn, B.; Pilon, L. Physical Interpretations of Nyquist Plots for EDLC Electrodes and Devices. *J. Phys. Chem. C* **2018**, *122* (1), 194–206. https://doi.org/10.1021/acs.jpcc.7b10582.

(11) Remita, E.; Boughrara, D.; Tribollet, B.; Vivier, V.; Sutter, E.; Ropital, F.; Kittel, J. Diffusion Impedance in a Thin-Layer Cell: Experimental and Theoretical Study on a Large-Disk Electrode. *J. Phys. Chem. C* **2008**, *112* (12), 4626–4634. https://doi.org/https://doi.org/10.1021/jp710407a.

(12) Hunter, T. B.; Tyler, P. S.; Smyrl, W. H.; White, H. S. Impedance Analysis of Poly(Vinylferrocene) Films: The Dependence of Diffusional Charge Transport and Exchange Current Density on Polymer Oxidation State. *J. Electrochem. Soc.* **1987**, *134* (9), 2198–2204. https://doi.org/10.1149/1.2100851.

(13) Shigyou, K.; Sun, L.; Yajima, R.; Takigaura, S.; Tajima, M.; Furusho, H.; Kikuchi, Y.; Miyazawa, K.; Fukuma, T.; Taoka, A.; Ando, T.; Watanabe, S. Geometrical Characterization of Glass Nanopipettes with Sub-10 Nm Pore Diameter by Transmission Electron Microscopy. *Anal. Chem.* **2020**, *92* (23), 15388–15393. https://doi.org/10.1021/acs.analchem.0c02884.

(14) Bae, J. H.; Wang, D.; Hu, K.; Mirkin, M. V. Surface-Charge Effects on Voltammetry in Carbon Nanocavities. *Anal. Chem.* **2019**, *91* (9), 5530–5536. https://doi.org/10.1021/acs.analchem.9b00426.

(15) Wang, Y.; Liu, R.; Shen, X.; Wang, D. Multivalent Ion-Modulated Electron Transfer Processes in Carbon Nanopipettes. *J. Phys. Chem. Lett.* **2022**, *13* (49), 11369. https://doi.org/10.1021/acs.jpclett.2c03322.

(16) Ramirez, P.; Cervera, J.; Nasir, S.; Ali, M.; Ensinger, W.; Mafe, S. Electrochemical Impedance Spectroscopy of Membranes with Nanofluidic Conical Pores. *J. Colloid Interface Sci.* **2024**, *655*, 876–885. https://doi.org/10.1016/j.jcis.2023.11.060.

(17) Lide, D. R. *CRC Handbook of Chemistry and Physics*, 90th Editi.; 2010.

(18) Yuan, X.-Z.; Song, C.; Wang, H.; Zhang, J. EIS Equivalent Circuits. In *Electrochemical Impedance Spectroscopy in PEM Fuel Cells*; Springer London: London, 2010; pp 139–192. https://doi.org/10.1007/978-1-84882-846-9_4.

(19) Lazanas, A. C.; Prodromidis, M. I. Electrochemical Impedance Spectroscopy—A Tutorial. *ACS Meas. Sci. Au* **2023**, *3* (3), 162–193. https://doi.org/10.1021/acsmeasuresciau.2c00070.

(20) Yang, H. J.; Han, D.; Kim, J.; Kim, Y. H.; Bae, J. H. Constant Phase Element Affected by Ion Transport in Nanoporous Electrodes. *J. Electroanal. Chem.* **2022**, *922*, 116766. https://doi.org/10.1016/j.jelechem.2022.116766.

(21) Robayo-Molina, I.; Crespo, G. A.; Cuartero, M. Usefulness of the Distribution of Relaxation Time Method in Electroanalytical Systems: The Case of Voltammetric Ion-Selective Electrodes. *ACS Omega* **2024**, *9* (7), 8162–8172. https://doi.org/10.1021/acsomega.3c08656.

(22) Yang, C.; Hu, K.; Wang, D.; Zubi, Y.; Lee, S. T.; Puthongkham, P.; Mirkin, M. V.; Venton, B. J. Cavity Carbon-Nanopipette Electrodes for Dopamine Detection. *Anal. Chem.* **2019**, *91* (7), 4618–4624. https://doi.org/10.1021/acs.analchem.8b05885.

Annex 1. Protocol for numerical simulations (COMSOL report)

Contents

1. Global Definitions……………………………………………………………………......

1.1. Parameters……………………………………………………………………………...

1.2. Shared Properties……………………………………………………………………….

2. Component 1 ………………………………………………………………………….

2.1. Definitions……………………………………………………………………………...

2.2. Geometry……………………………………………………………………………….

2.3. Materials……………………………………………………………………………..

2.4. Electroanalysis………………………………………………………………………….

2.5. Mesh 2………………………………………………………………………………..

3. Study 1……………………………………………………………………………………

3.1. Parametric Sweep………………………………………………………………………

3.2. Cyclic Voltammetry……………………………………………………………………

3.3. Solver Configurations ………………………………………………………………...

4. Results…………………………………………………………………………………..

4.1. Data Sets……………………………………………………………………………….

4.2. Plot Groups…………………………………………………………………………….

1. Definitions

1.1 Parameters

PARAMETERS 1

Name	Expression	Value	Description
v	0.05[V/s]	0.05 V/s	Voltammetric scan rate
c_bulk	0.77[mmol/L]	0.77 mol/m³	Reactant concentration
DA	7.3e-10[m^2/s]	7.3E−10 m²/s	Reactant diffusion coefficient (Ferro)
DB	7.3e-10[m^2/s]	7.3E−10 m²/s	Product diffusion coefficient (Ferri)
k0	10 [cm/s]	0.1 m/s	Reaction rate
E_vertex1	-0.2[V]	−0.2 V	Start potential
E_vertex2	0.7[V]	0.7 V	Upper potential
a1	80 [nm]	8E−8 m	CNP radius
L_CNP	45 [um]	4.5E−5 m	Solution depth
a2	9000 [nm]	9E−6 m	Higher radius
a3	30 [nm]	3E−8 m	Carbon layer
a4	100 [nm]	1E−7 m	Glass layer
c_Cl	0.3 [mol/l]	300 mol/m³	Chloride concentration
c_K	c_Cl + (4*c_bulk)	303.08 mol/m³	Potassium concentration
tH	(E_vertex2 - E_vertex1)*2/v	36 s	Analysis time
λ	0.0075 [Sm²/mol]	0.00075 Sm²/mol	K^+ and Cl^- conductivity

1.2 Shared Properties

1.3 Model entry

Tag	cminpt

2 Component 1

SETTINGS

Description	Value
Unit system	Same as global system (SI)
Avoid inverted elements by curving interior domain elements	Off

2.1 Definitions

2.2 Coordinate Systems

Boundary system 1

Coordinate system type	Boundary system
Tag	sys1

COORDINATE NAMES

First	Second	Third
t1	to	n

2.3 Geometry

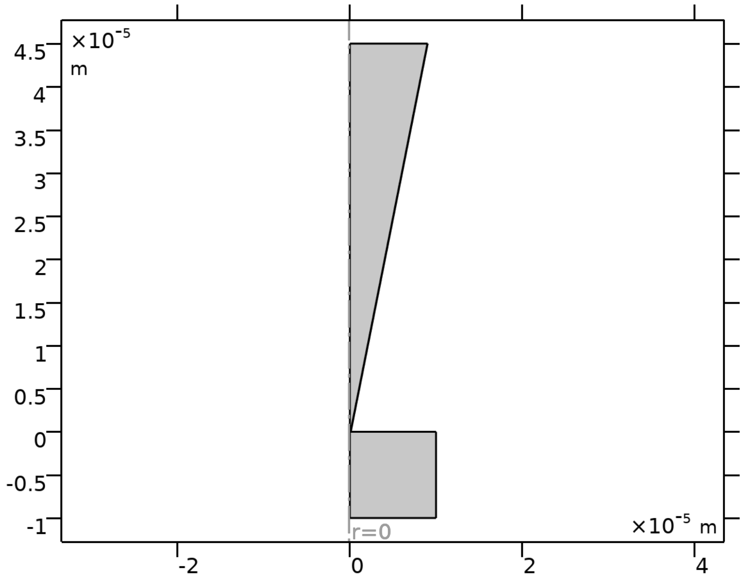

Figure A1

Geometry 2

UNITS

Length unit	m
Angular unit	deg

GEOMETRY STATISTICS

Description	Value
Space dimension	2
Number of domains	3
Number of boundaries	12
Number of vertices	10

2.4 Materials

2.4.1 KCl 0.3 M

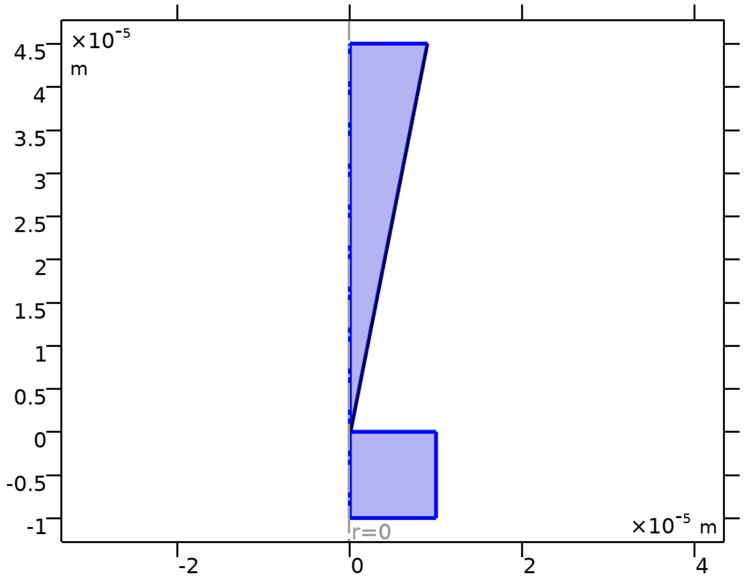

Figure A2

KCl 0.3 M

SELECTION

Geometric entity level	Domain
Selection	Geometry geom2: Dimension 2: Domains 1–2

BASIC

**Description**	**Value**	**Unit**
Dynamic viscosity	eta(T)	Pa·s
Ratio of specific heats	1	1
Electrical conductivity	5.5E-6	S/m
Heat capacity at constant pressure	Cp(T)	J/(kg·K)
Density	rho(T)	kg/m³
Thermal conductivity	k(T)	W/(m·K)
Speed of sound	cs(T)	m/s

ELECTROLYTE CONDUCTIVITY

**Description**	**Value**	**Unit**
Electrolyte conductivity	1.288	S/m

2.4.2 Graphite 1

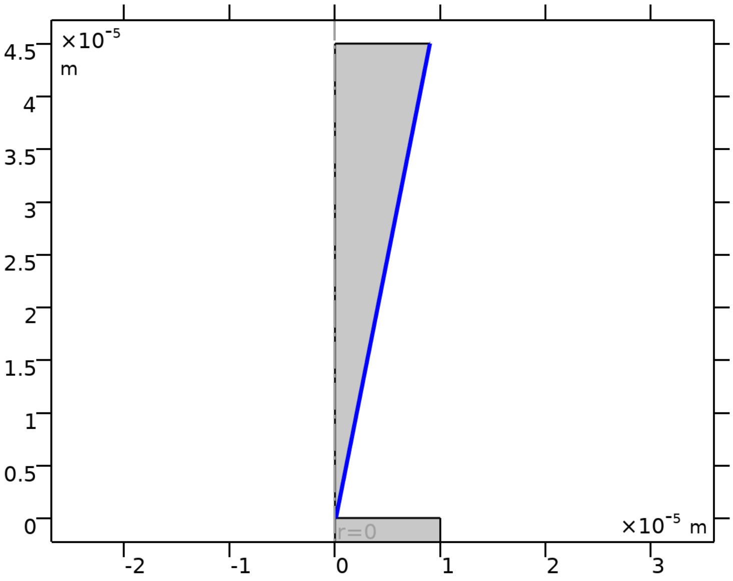

Figure A3

Graphite 1

SELECTION

Geometric entity level	Domain
Selection	Geometry geom2: Dimension 2: Domain 3

BASIC

Description	Value	Unit
Relative permeability	1	1
Electrical conductivity	3000	S/m
Heat capacity at constant pressure	710	J/(kg·K)
Relative permittivity	1	1
Surface emissivity	1	1
Density	1950	kg/m³
Thermal conductivity	45000/T	W/(m·K)

CONDUCTIVIDAD DE ELECTROLITO

Description	Value	Unit
Electrolyte conductivity	1	S/m

2.5 Electroanalysis

USED PRODUCTS

COMSOL Multiphysics
Corrosion Module

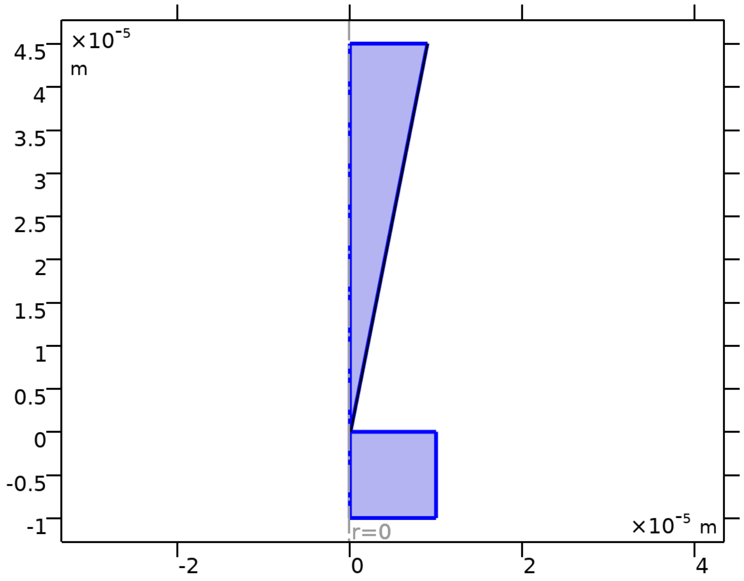

Figure A4

Electroanalysis

SELECTION

Geometric entity level	Domain
Selection	Geometry geom2: Dimension 2: Domains 1–2

EQUATIONS

$$\frac{\partial c_i}{\partial t} + \nabla \cdot \mathbf{J}_i + \mathbf{u} \cdot \nabla c_i = R_i$$

$$\nabla \cdot \mathbf{i}_l = F \sum_i z_i R_i + Q_l$$

$$\nabla \cdot \mathbf{i}_s = -F \sum_i z_i R_i + Q_s$$

$$\mathbf{J}_i = -D_i \nabla c_i - z_i u_{m,i} F c_i \nabla \phi_l$$

$$\mathbf{i}_l = -\sigma_l \nabla \phi_l$$

$$\mathbf{i}_s = -\sigma_s \nabla \phi_s$$

$$\phi_l = \mathrm{phil}, \quad \phi_s = \mathrm{phis}$$

2.5.1 Interface Settings

Discretization

SETTINGS

**Description**	**Value**
Concentration	Linear
Electrolyte potential	Quadratic
Electric potential	Quadratic

SETTINGS

**Description**	**Value**
Equation form	Study controlled

Electrolyte Charge Conservation

SETTINGS

Description	Value
Charge conservation model	Supporting electrolyte

Species Activity

SETTINGS

Description	Value
Species activity	Ideal

Physics vs. Materials Reference Electrode Potential

SETTINGS

Description	Value
Physics vs. materials reference electrode potential	0 V

2.5.2 Species Charges 1

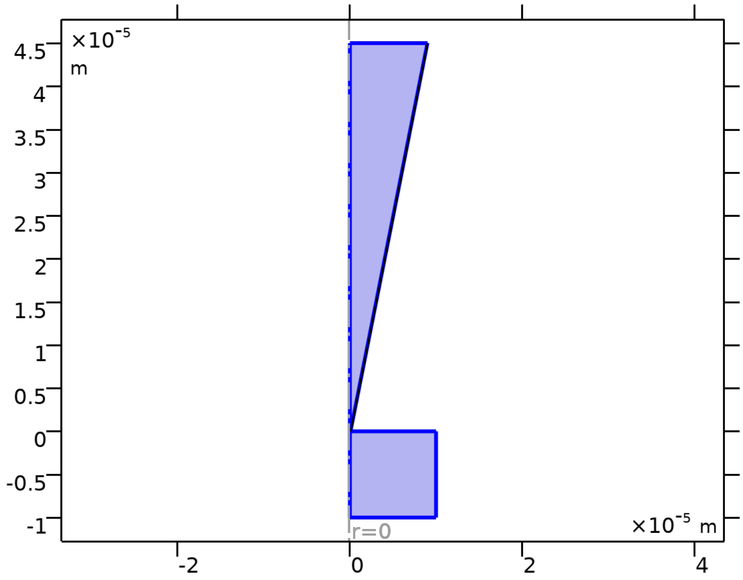

Figure A5

Species Charges 1

SELECTION

Geometric entity level	Domain
Selection	Geometry geom2: Dimension 2: All domains

Charge

SETTINGS

Description	Value
Charge number	{1, -1, -4, -3}

Model Input

SETTINGS

Description	Value
Temperature	Common model input

Variables

Name	Expression	Unit	Description	Selection
tcd.z_cK	1	1	Charge number	Domains 1–2
tcd.z_cCl	-1	1	Charge number	Domains 1–2
tcd.z_cA	-4	1	Charge number	Domains 1–2

Name	Expression	Unit	Description	Selection
tcd.z_cB	-3	1	Charge number	Domains 1–2

2.5.3 Electrolyte

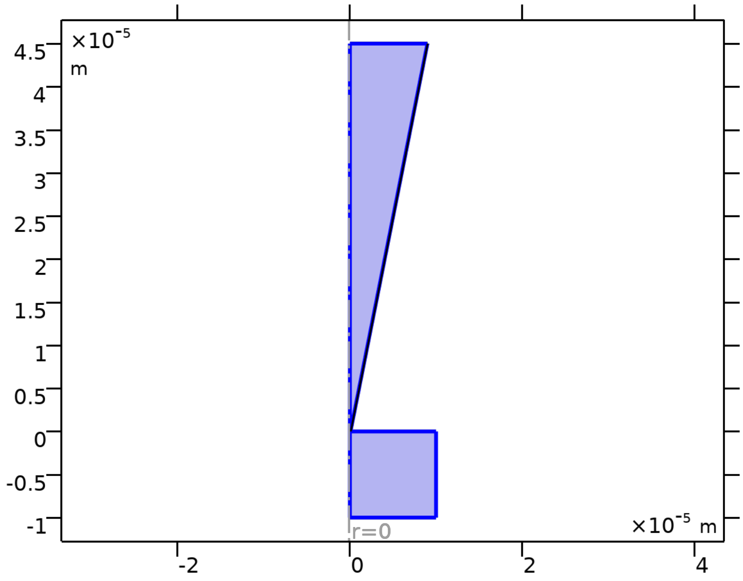

Figure A6

Electrolyte

SELECTION

Geometric entity level	Domain
Selection	Geometry geom2: Dimension 2: All domains

EQUATIONS

$$\frac{\partial c_i}{\partial t} + \nabla \cdot \mathbf{J}_i + \mathbf{u} \cdot \nabla c_i = R_i$$

$$\nabla \cdot \mathbf{i}_l = F \sum_i z_i R_i + Q_l$$

$$\mathbf{J}_i = -D_i \nabla c_i - z_i u_{m,i} F c_i \nabla \phi_l$$

$$\mathbf{i}_l = -\sigma_l \nabla \phi_l$$

Convection

SETTINGS

Description	Value	Unit
Velocity field	User defined	
Velocity field	{0, 0, 0}	m/s

Diffusion

SETTINGS

Description	Value	Unit
Material	KCl 0.3 M (mat2)	
Diffusion coefficient	User defined	
Diffusion coefficient	2.029E-9	m²/s
Diffusion coefficient	User defined	
Diffusion coefficient	2.029E-9	m²/s
Diffusion coefficient	User defined	
Diffusion coefficient	DA	m²/s

Description	Value	Unit
Diffusion coefficient	User defined	
Diffusion coefficient	DB	m²/s

Migration in Electric Field

SETTINGS

Description	Value
Mobility	Nernst - Einstein relation

Solvent

SETTINGS

Description	Value	Unit
Electrolyte conductivity	User defined	
Electrolyte conductivity	λ*(cK + cCl)	S/m

Coordinate System Selection

SETTINGS

Description	Value
Coordinate system	Global coordinate system

Model Input

SETTINGS

Description	Value
Temperature	Common model input

Shape functions

Name	Shape function	Unit	Description	Shape frame	Selection
cK	Lagrange (Linear)	mol/m³	Concentration	Material	Domains 1–2
cCl	Lagrange (Linear)	mol/m³	Concentration	Material	Domains 1–2
cA	Lagrange (Linear)	mol/m³	Concentration	Material	Domains 1–2
cB	Lagrange (Linear)	mol/m³	Concentration	Material	Domains 1–2
phil	Lagrange (Quadratic)	V	Electrolyte potential	Material	Domains 1–2

Weak Expressions

Weak expression	Integration order	Integration frame	Selection
2*(tcd.Ilr*test(philr)+tcd.Ilz*test(philz)+tcd.Qli*test(phil))*tcd.d*pi*r	2	Material	Domains 1–2
-2*(tcd.u*cKr+tcd.w*cKz)*test(cK)*(isScalingSystemDomain==0)*tcd.d*pi*r	2	Material	Domains 1–2
2*tcd.cbf_cK*test(cK)*tcd.d*pi*r	2	Material	Boundaries 1–10
-2*(tcd.u*cClr+tcd.w*cClz)*test(cCl)*(isScalingSystemDomain==0)*tcd.d*pi*r	2	Material	Domains 1–2
2*tcd.cbf_cCl*test(cCl)*tcd.d*pi*r	2	Material	Boundaries 1–10

Weak expression	Integration order	Integration frame	Selection
-2*(tcd.u*cAr+tcd.w*cAz)*test(cA)*(isScalingSystemDomain==0)*tcd.d*pi*r	2	Material	Domains 1–2
2*tcd.cbf_cA*test(cA)*tcd.d*pi*r	2	Material	Boundaries 1–10
-2*(tcd.u*cBr+tcd.w*cBz)*test(cB)*(isScalingSystemDomain==0)*tcd.d*pi*r	2	Material	Domains 1–2
2*tcd.cbf_cB*test(cB)*tcd.d*pi*r	2	Material	Boundaries 1–10
2*tcd.z_cK*F_const*cK*((-tcd.um_cKrr*d(tcd.V,r)-tcd.um_cKrz*d(tcd.V,z))*test(cKr)+(-tcd.um_cKzr*d(tcd.V,r)-tcd.um_cKzz*d(tcd.V,z))*test(cKz))*tcd.d*pi*r	2	Material	Domains 1–2
2*tcd.z_cCl*F_const*cCl*((-tcd.um_cClrr*d(tcd.V,r)-tcd.um_cClrz*d(tcd.V,z))*test(cClr)+(-tcd.um_cClzr*d(tcd.V,r)-tcd.um_cClzz*d(tcd.V,z))*test(cClz))*tcd.d*pi*r	2	Material	Domains 1–2
2*tcd.z_cA*F_const*cA*((-tcd.um_cArr*d(tcd.V,r)-tcd.um_cArz*d(tcd.V,z))*test(cAr)+(-tcd.um_cAzr*d(tcd.V,r)-tcd.um_cAzz*d(tcd.V,z))*test(cAz))*tcd.d*pi*r	2	Material	Domains 1–2
2*tcd.z_cB*F_const*cB*((-tcd.um_cBrr*d(tcd.V,r)-tcd.um_cBrz*d(tcd.V,z))*test(cBr)+(-tcd.um_cBzr*d(tcd.V,r)-tcd.um_cBzz*d(tcd.V,z))*test(cBz))*tcd.d*pi*r	2	Material	Domains 1–2
2*(-d(tcd.epsilon_p*cK,t)*test(cK)+tcd.dflux_cKr*test(cKr)+tcd.dflux_cKz*test(cKz))*tcd.d*pi*r	2	Material	Domains 1–2
2*(-d(tcd.epsilon_p*cCl,t)*test(cCl)+tcd.dflux_cClr*test(cClr)+tcd.dflux_cClz*test(cClz))*tcd.d*pi*r	2	Material	Domains 1–2
2*(-d(tcd.epsilon_p*cA,t)*test(cA)+tcd.dflux_cAr*test(cAr)+tcd.dflux_cAz*test(cAz))*tcd.d*pi*r	2	Material	Domains 1–2
2*(-d(tcd.epsilon_p*cB,t)*test(cB)+tcd.dflux_cBr*test(cBr)+tcd	2	Material	Domains 1–2

Weak expression	Integration order	Integration frame	Selection
.dflux_cBz*test(cBz))*tcd.d*pi*r			
2*tcd.streamline*(isScalingSystemDomain==0)*tcd.d*pi*r	2	Material	Domains 1–2
2*tcd.crosswind*(isScalingSystemDomain==0)*tcd.d*pi*r	4	Material	Domains 1–2

2.5.4 Axial symmetry

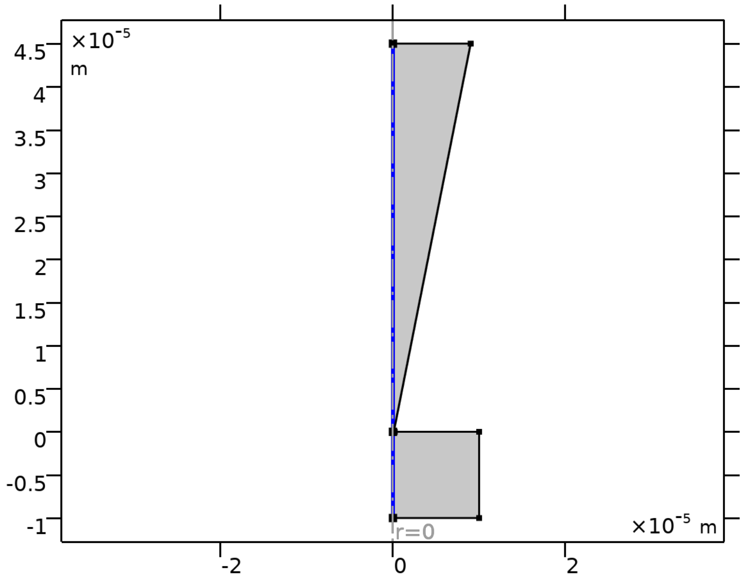

Figure A7

Axial symmetry

SELECTION

Geometric entity level	Boundary
Selection	Geometry geom2: Dimension 1: All boundaries

2.5.5 No flux

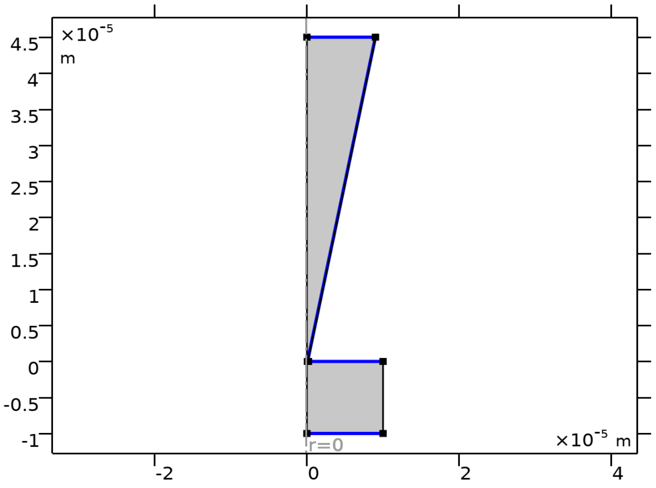

Figure A8

No flux 1

SELECTION

Geometric entity level	Boundary
Selection	Geometry geom2: Dimension 1: All boundaries

EQUATIONS

$$-\mathbf{n}\cdot\mathbf{J}_i = 0$$

Convection

SETTINGS

**Description**	**Value**
Include	Off

2.5.6 Isolation 1

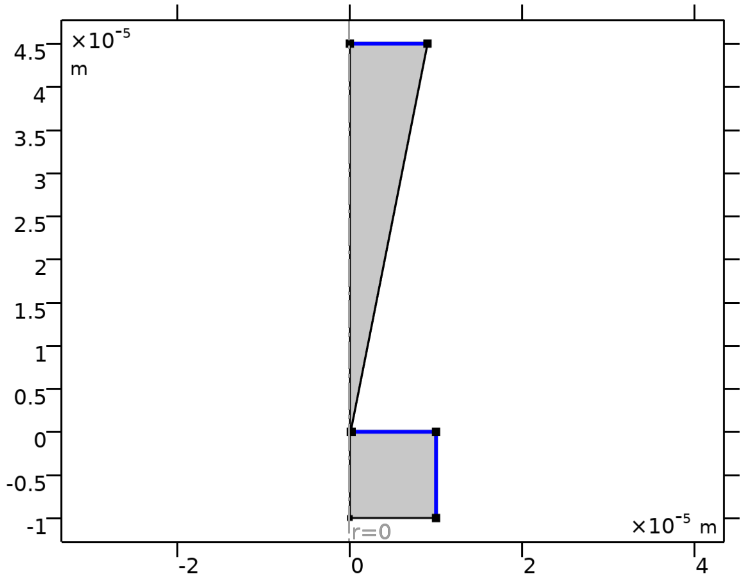

Figure A9

Isolation 1

SELECTION

Geometric entity level	Boundary
Selection	Geometry geom2: Dimension 1: All boundaries

EQUATIONS

$$-\mathbf{n}\cdot\mathbf{i}_l = 0, \quad -\mathbf{n}\cdot\mathbf{i}_s = 0$$

2.5.7 Initial values 1

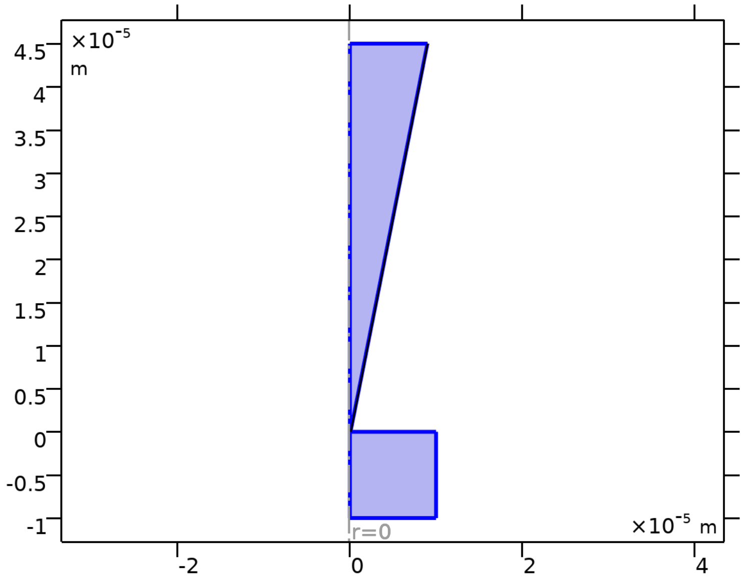

Figure A10

Initial values 1

SELECTION

Geometric entity level	Domain
Selection	Geometry geom2: Dimension 2: All domains

Initial Values

SETTINGS

**Description**	**Value**	**Unit**
Concentration	{c_K, c_Cl, c_bulk, 0}	mol/m³
Electrolyte potential	0	V
Electric potential	0	V

2.5.8 Electrolyte potential 1

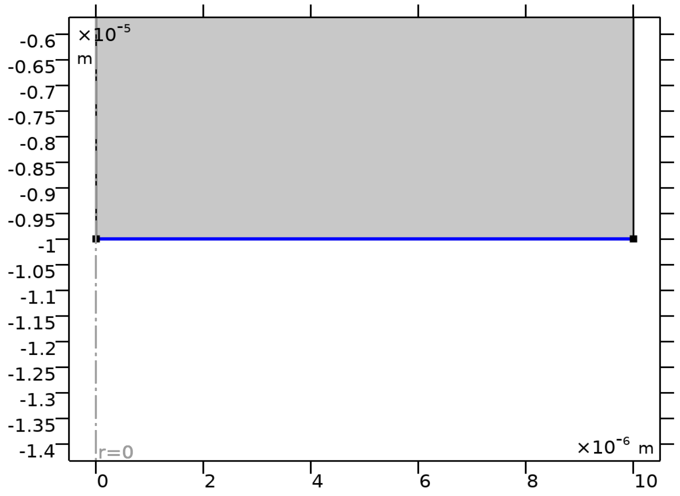

Figure A11

Electrolyte potential 1

SELECTION

Geometric entity level	Boundary
Selection	Geometry geom2: Dimension 1: Boundary 2

EQUATIONS

$$\phi_l = \phi_{l,bnd}$$

Electrolyte Potential

SETTINGS

Description	Value	Unit
Boundary electrolyte potential	0	V

Variables

Name	Expression	Unit	Description	Selection
tcd.philbnd	0[V]	V	Boundary electrolyte potential	Boundary 2

Constraints

Constraint	Constraint force	Shape function	Selection	Details
tcd.philbnd-phil	test(tcd.philbnd-phil)	Lagrange (Quadratic)	Boundary 2	Elemental

2.5.9 Concentration 1

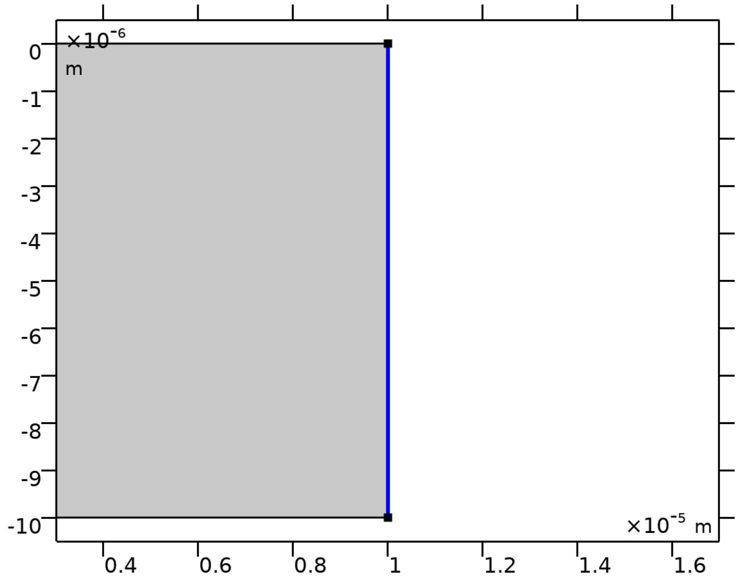

Figure A12

Concentration 1

SELECTION

Geometric entity level	Boundary
Selection	Geometry geom2: Dimension 1: Boundary 5

EQUATIONS

$$c_i = c_{0,i}$$

Concentration

SETTINGS

Description	Value	Unit
Species cK	On	
Species cCl	On	
Species cA	On	
Species cB	On	
Concentration	{c_K, c_Cl, c_bulk, 0}	mol/m³

Variables

Name	Expression	Unit	Description	Selection
tcd.c0_cK	c_K	mol/m³	Concentration	Boundary 5
tcd.c0_cCl	c_Cl	mol/m³	Concentration	Boundary 5
tcd.c0_cA	c_bulk	mol/m³	Concentration	Boundary 5
tcd.c0_cB	0	mol/m³	Concentration	Boundary 5

Constraints

Constraint	Constraint force	Shape function	Selection	Details
-tcd.cVar_cK+tcd.c0_cK	test(-tcd.cVar_cK+tcd.c0_cK)	Lagrange (Linear)	Boundary 5	Elemental
-tcd.cVar_cCl+tcd.c0_cCl	test(-tcd.cVar_cCl+tcd.c0_cCl)	Lagrange (Linear)	Boundary 5	Elemental
-tcd.cVar_cA+tcd.c0_cA	test(-tcd.cVar_cA+tcd.c0_cA)	Lagrange (Linear)	Boundary 5	Elemental
-tcd.cVar_cB+tcd.c0_cB	test(-tcd.cVar_cB+tcd.c0_cB)	Lagrange (Linear)	Boundary 5	Elemental

2.5.10 Electrode surface 1

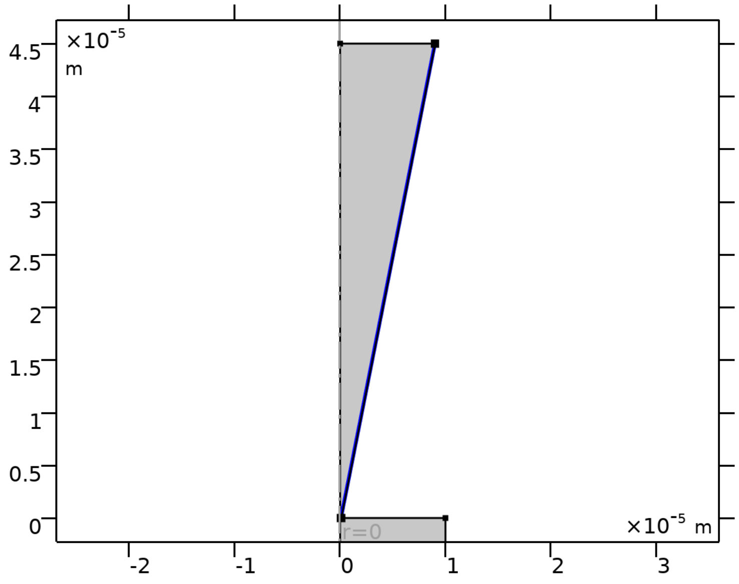

Figure A13

Electrode surface 1

SELECTION

Geometric entity level	Boundary
Selection	Geometry geom2: Dimension 1: Boundaries 8–9

EQUATIONS

$$\mathbf{n}\cdot\mathbf{i}_l = i_{total}$$
$$i_{total} = \sum_m i_{loc,m} + i_{dl}$$
$$-\mathbf{n}\cdot\mathbf{J}_i = R_{i,tot}, \quad R_{i,tot} = \sum_m R_{i,m} + R_{dl,i}$$
....

Dissolving-Depositing Species

SETTINGS

Description	Value
Species	
Solve for surface concentration variables	On

Adsorbing-Desorbing Species

SETTINGS

Description	Value	Unit
Density of sites	1E-5	mol/m²
Adsorbing-desorbing species		

Species	Site occupancy number
1	

Film Resistance

SETTINGS

Description	Value
Film resistance	No film resistance

Harmonic Perturbation

SETTINGS

Description	Value	Unit
Perturbation amplitude	0	V

Electrode Phase Potential Condition

SETTINGS

Description	Value	Unit
Electrode phase potential condition	Cyclic voltammetry	
Linear sweep rate	v	V/s
Start potential	On	
Start potential	E_vertex1	V
Number of cycles	1	1
Vertex potential 1	E_vertex1	V
Vertex potential 2	E_vertex2	V
End potential	Off	

Variables

Name	Expression	Unit	Description	Selection	Details
tcd.nil	tcd.itot	A/m²	Inward electrolyte current density	Boundaries 8–9	+ operation
tcd.mulstopcond	t>tcd.ncycle_es1*abs(2*(E_vertex1-E_vertex2))/v	1	Multiplicative stop condition	Global	* operation
tcd.Ect	tcd.phisext-phil	V	Electrode potential	Boundaries 8–9	
tcd.Gamma_es1	1.0E-5[mol/m^2]	mol/m²	Density of sites	Boundaries 8–9	

Name	Expression	Unit	Description	Selection	Details
tcd.ncycle_es1	1	1	Number of cycles	Global	
tcd.itot	0	A/m²	Total interface current density	Boundaries 8–9	+ operation
tcd.phis_es1	tcd.es1.int(tcd.phisext*tcd.dvolfactor*tcd.d)/tcd.Area_es1	V	Electric potential	Global	
tcd.phisext	tcd.cv_es1(t)	V	External electric potential	Boundaries 8–9	
tcd.dvolfactor	2*pi*r	m	Differential volume factor	Boundaries 8–9	Meta
tcd.Area_es1	tcd.es1.int(tcd.dvolfactor*tcd.d)	m²	Area	Global	
tcd.itotavg_es1	tcd.es1.int(tcd.itot*tcd.dvolfactor*tcd.d)/tcd.Area_es1	A/m²	Average total interface current density	Global	
tcd.Temp	tcd.es1.minput_temperature	K	Temperature	Boundaries 8–9	
tcd.Evsref	tcd.phisext-phil	V	Electrode potential vs. adjacent reference	Boundaries 8–9	
tcd.Ectmat	tcd.Ect	V	Electrode potential	Boundaries 8–9	
tcd.Itot_es1	tcd.es1.int(tcd.itot*tcd.dvolfactor*tcd.d)	A	Total current	Global	
tcd.rhos	8960	kg/m³	Density	Boundaries 8–9	
tcd.Ms	0.06355	kg/mol	Molar mass	Boundaries 8–9	
tcd.Sigma	1	1	Site occupancy number	Boundaries 8–9	
tcd.cycle_es1	max(round(0.5+min(t,(-1+tcd.ncycle_es1)*abs(2*(E_vertex1-E_vertex2))/v)*v/abs(2*(E_vertex1-E_vertex2))),1)	1	Cycle number	Global	

Electrode reaction 1

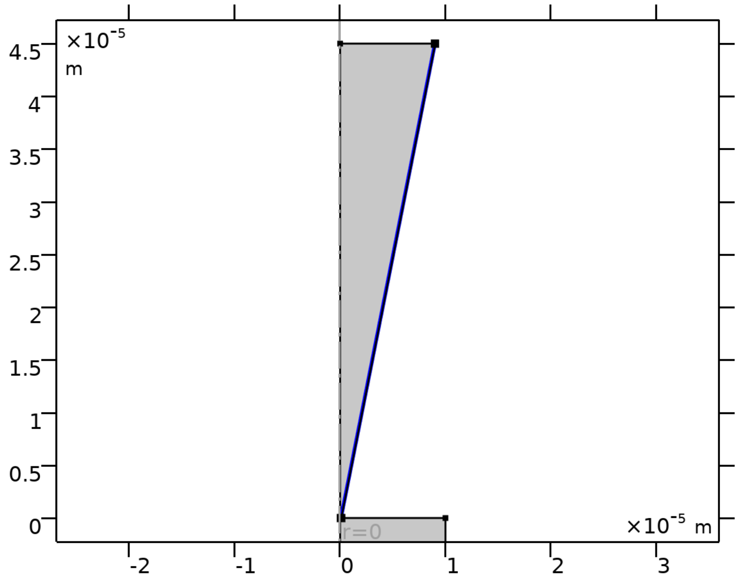

Figure A14

Electrode reaction 1

SELECTION

Geometric entity level	Boundary
Selection	Geometry geom2: Dimension 1: All boundaries

EQUATIONS

$$\eta = E_{ct} - E_{eq}, \quad E_{ct} = \phi_{s,ext} - \phi_l$$

$$R_i = \frac{-\nu_i i_{loc}}{nF}$$

Stoichiometric Coefficients

SETTINGS

Description	Value
Number of participating electrons	1
Stoichiometric coefficient	{0, 0, 1, -1}

Equilibrium Potential

SETTINGS

Description	Value	Unit
Equilibrium potential	Nernst equation	
Reference equilibrium potential	0.2	V

Reference Concentrations

Electrolyte species	Reference concentrations
cK	1[M]
cCl	1[M]
cA	1[M]
cB	1[M]

Electrode Kinetics

SETTINGS

Description	Value	Unit
Local current density expression	From kinetics expression	

Description	Value	Unit
Kinetics expression type	Butler - Volmer	
Exchange current density type	Mass action law	
Reference exchange current density	1000*F_const	A/m²
Anodic transfer coefficient	0.5	1
Limiting current density	Off	
Linearize concentration dependence for low concentrations	On	
Concentration linearization limit	0.001	mol/m³

Heat of Reaction

SETTINGS

Description	Value	Unit
Specify	Temperature derivative	
Temperature derivative of equilibrium potential	User defined	
Temperature derivative of equilibrium potential	0	V/K

Model Input

SETTINGS

Description	Value
Temperature	Common model input

Weak Expressions

Weak expression	Integration order	Integration frame	Selection
2*tcd.es1.er1.N0_cK*test(cK) *tcd.d*pi*r	2	Material	Boundaries 8–9
2*tcd.es1.er1.N0_cCl*test(cCl)*tcd.d*pi*r	2	Material	Boundaries 8–9
2*tcd.es1.er1.N0_cA*test(cA) *tcd.d*pi*r	2	Material	Boundaries 8–9
2*tcd.es1.er1.N0_cB*test(cB) *tcd.d*pi*r	2	Material	Boundaries 8–9
2*tcd.iloc_er1*tcd.d*test(phil)* pi*r	2	Material	Boundaries 8–9

Double-layer capacitance 1

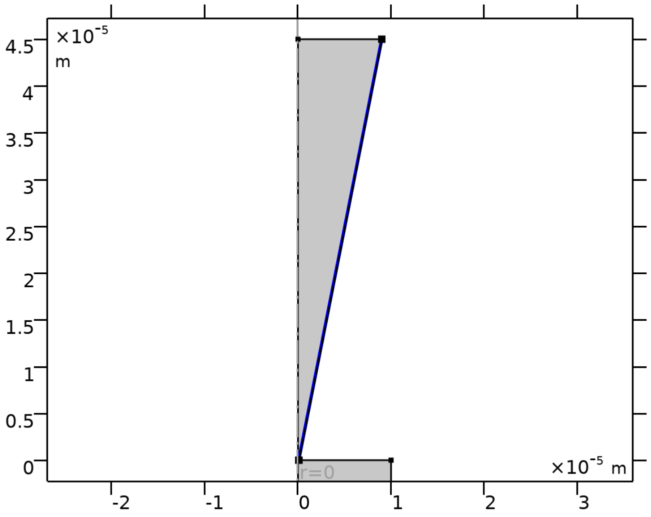

Figure A15

Double-layer capacitance 1

SELECTION

Geometric entity level	Boundary
Selection	Geometry geom2: Dimension 1: All boundaries

EQUATIONS

$$i_{\mathrm{dl}} = \left(\frac{\partial(\phi_{\mathrm{s,ext}} - \phi_{\mathrm{l}})}{\partial t}\right) C_{\mathrm{dl}}$$

$$R_{\mathrm{dl},i} = \frac{-\nu_i i_{\mathrm{dl}}}{nF}$$

Double-Layer Capacitance

SETTINGS

Description	Value	Unit
Electrical double layer capacitance	0.2	F/m²

Stoichiometric Coefficients

SETTINGS

Description	Value
Number of participating electrons	1
Stoichiometric coefficient	{1, -1, 1, -1}

Variables

Name	Expression	Unit	Description	Selection	Details
tcd.itot	tcd.idl	A/m²	Total interface current density	Boundaries 8–9	+ operation
tcd.Cdl	0.2[F/m^2]	F/m²	Electrical double layer capacitance	Boundaries 8–9	
tcd.idl	d(tcd.Ect,t)*tcd.Cdl	A/m²	Double-layer current density	Boundaries 8–9	
tcd.es1.dlc1.N0_cK	-tcd.idl/F_const	mol/(m²·s)	Inward flux	Boundaries 8–9	
tcd.es1.dlc1.N0_cCl	tcd.idl/F_const	mol/(m²·s)	Inward flux	Boundaries 8–9	
tcd.es1.dlc1.N0_cA	-tcd.idl/F_const	mol/(m²·s)	Inward flux	Boundaries 8–9	
tcd.es1.dlc1.N0_cB	tcd.idl/F_const	mol/(m²·s)	Inward flux	Boundaries 8–9	

Weak Expressions

Weak expression	Integration order	Integration frame	Selection
2*tcd.idl*tcd.d*test(phil)*pi*r	2	Material	Boundaries 8–9
2*tcd.es1.dlc1.N0_cK*test(cK)*tcd.d*pi*r	2	Material	Boundaries 8–9
2*tcd.es1.dlc1.N0_cCl*test(cCl)*tcd.d*pi*r	2	Material	Boundaries 8–9
2*tcd.es1.dlc1.N0_cA*test(cA)*tcd.d*pi*r	2	Material	Boundaries 8–9
2*tcd.es1.dlc1.N0_cB*test(cB)*tcd.d*pi*r	2	Material	Boundaries 8–9

2.6 Mesh

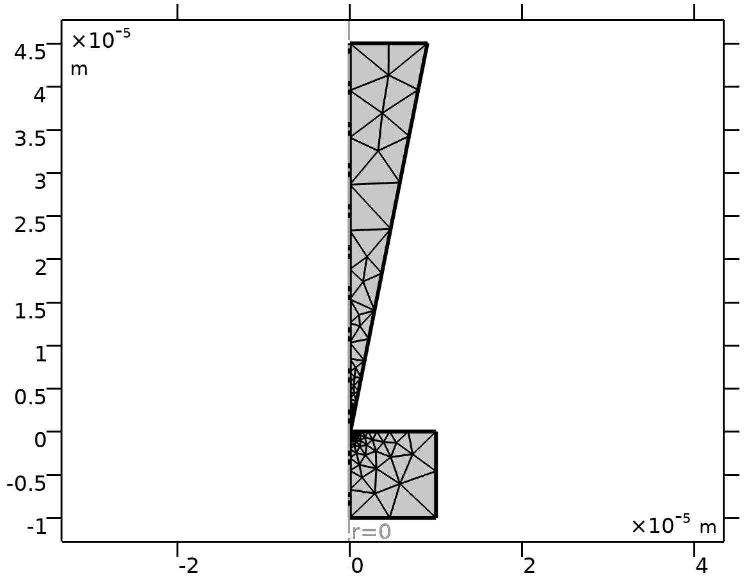

Figure A16

Mesh

2.6.1 Size

SETTINGS

**Description**	**Value**
Maximum element size	5.5E-6
Minimum element size	1.1E-7
Curvature factor	0.4
Maximum element growth rate	1.4
Predefined size	Coarse

2.6.2 Free Triangular 1 (ftri1)

SELECTION

Geometric entity level	Domain
Selection	Geometry geom2: Dimension 2: Domains 1–2

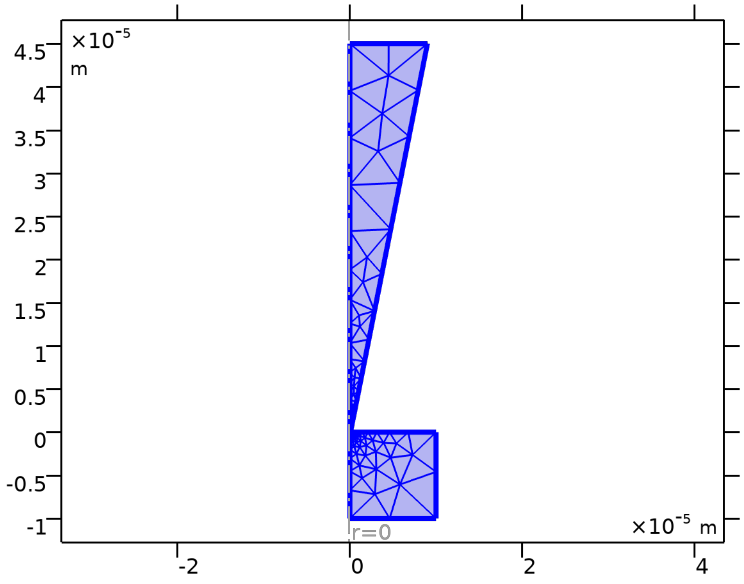

Figure A17

SETTINGS

Description	Value
Number of iterations	4
Maximum element depth to process	4

3 Study 1

COMPUTATION INFORMATION

Computation time	5 min 40 s

3.1 Parametric Sweep

Parameter name	Parameter value list	Parameter unit
v	0.25,0.05,0.005,0.001	V/s

STUDY SETTINGS

Description	Value
Sweep type	Specified combinations
Parameter name	v
Unit	V/s

PARAMETERS

Parameter name	Parameter value list	Parameter unit
v (Voltammetric scan rate)	0.25,0.05,0.005,0.001	V/s

3.2 Cyclic Voltammetry

STUDY SETTINGS

Description	Value
Include geometric nonlinearity	Off

PHYSICS AND VARIABLES SELECTION

Physics interface	Solve for	Equation form
Electroanalysis (tcd)	On	Automatic (Time dependent)

STORE IN OUTPUT

Interface	Output	Selection
Electroanalysis (tcd)	Physics controlled	

MESH SELECTION

Component	Mesh
Componente 1	Malla 2

3.3 Solver Configurations

3.3.1 Solution 1

Compile Equations: Cyclic Voltammetry (st1)

STUDY AND STEP

Description	Value
Use study	Study 1
Use study step	Cyclic Voltammetry

Dependent Variables 1 (v1)

GENERAL

Description	Value
Defined by study step	Step 1: Cyclic Voltammetry

INITIAL VALUE CALCULATION CONSTANTS

Description	Value
Parameters	Manual

INITIAL VALUE CALCULATION CONSTANTS

Constant name	Initial value source
t	{0 tH+1}[s]
timestep	min(1e100[s],1e-4[V]/abs(v))

Concentration (comp1.cA) (comp1_cA)

GENERAL

Description	Value
Field components	comp1.cA
Internal variables	{comp1.uflux.cA, comp1.dflux.cA, comp1.tcd.dt2Inv_cA}

Concentration (comp1.cB) (comp1_cB)

GENERAL

Description	Value
Field components	comp1.cB
Internal variables	{comp1.uflux.cB, comp1.dflux.cB, comp1.tcd.dt2Inv_cB}

Electrolyte potential (comp1.phil) (comp1_phil)

GENERAL

Description	Value
Field components	comp1.phil
Internal variables	{comp1.uflux.phil, comp1.dflux.phil}

Concentration (comp1.cK) (comp1_cK)

GENERAL

Description	Value
Field components	comp1.cK
Internal variables	{comp1.uflux.cK, comp1.dflux.cK, comp1.tcd.dt2Inv_cK}

Concentration (comp1.cCl) (comp1_cCl)

GENERAL

Description	Value
Field components	comp1.cCl
Internal variables	{comp1.uflux.cCl, comp1.dflux.cCl, comp1.tcd.dt2Inv_cCl}

Time-Dependent Solver 1 (t1)

ABSOLUTE TOLERANCE

Description	Value
Tolerance method	Manual

ABSOLUTE TOLERANCE

Field	Method	Tolerance method	Tolerance factor	Derivative tolerance method	Time derivative factor	Tolerance	Tolerance for time derivatives
Concentration (comp1.cA)	Use global	Manual	0.1	Automatic	1	0.001	0.001
Concentration (comp1.cB)	Use global	Manual	0.1	Automatic	1	0.001	0.001

Field	Method	Tolerance method	Tolerance factor	Derivative tolerance method	Time derivative factor	Tolerance	Tolerance for time derivatives
Electrolyte potential (comp1.phil)	Use global	Manual	0.1	Automatic	1	0.001	0.001
Concentration (comp1.cK)	Use global	Manual	0.1	Automatic	1	0.001	0.001
Concentration (comp1.cCl)	Use global	Manual	0.1	Automatic	1	0.001	0.001

TIME STEPPING

Description	Value
Initial step	min(1e100[s], 1e-5[V]/abs(v))
Initial step	On
Maximum step constraint	Constant
Maximum step	min(1e100[s], abs((E_vertex1 - E_vertex2)/v))
Maximum BDF order	2

RESULTS WHILE SOLVING

Description	Value
Plot	On
Plot group	Voltamogramas cíclicos (elan)
Update at	Time steps taken by solver

Stop Condition 1 (st1)

STOP EXPRESSIONS

Stop expression	Stop if	Active	Description
elan.mulstopcond	True (>=1)	Off	elan (Cyclic voltammetry)

OUTPUT AT STOP

Description	Value
Add solution	Steps before and after stop
Add warning	Off

Fully Coupled 1 (fc1)

GENERAL

Description	Value
Linear solver	Direct 1

METHOD AND TERMINATION

Description	Value
Jacobian update	Once per time step
Maximum number of iterations	5

Direct 1 (d1)

GENERAL

Description	Value
Pivot threshold	0.1
Out-of-core	Off

ERROR

Description	Value
Factor in error estimate	400
